# Colors, characters, locations, and shapes: The capacity of working memory for multiple, dissimilar sets of items

**DOI:** 10.3758/s13421-025-01809-7

**Published:** 2026-01-14

**Authors:** Luísa Superbia-Guimarães, Nelson Cowan

**Affiliations:** 1 School of Psychology, University of Leeds, University Road, Woodhouse, Leeds, LS2 9JT UK; 2 Department of Psychological Sciences, University of Missouri, 210 McAlester Hall, Columbia, MO 65211 USA

**Keywords:** Working memory, Attention, Capacity, Similarity

## Abstract

Working memory (WM) often includes heterogenous items, as when one uses it while assembling a desk from sets of boards, knobs, bolts, and washers. Here, we investigate how WM capacity is limited when recalling multiple sets of items, for which performance surpasses the usual limits observed in single-set procedures. We presented participants (*N* = 181) with up to four sets of items for serial recall, usually of different stimulus types in the same trial (colors, characters, locations, and/or shapes). Conditions differed in the total number of items, the number of sets, and/or item types across sets in a trial. For uniformity in analyses, Set 1 was kept constant at three items of a type and was usually recalled first, free of output interference. In Experiment 1, recall of Set 1 was not only limited by the total number of items but also by the number of sets in a trial. Experiment 2 ruled out interference as an alternative explanation. Experiments 3–4 showed the dependency of the results on clearly grouped presentation of the sets. The results suggest that groups of items are associated as newly formed, often incomplete chunks offloaded from the focus of attention (FoA) to an activated portion of long-term memory (aLTM) for later retrieval. This offloading process would spare capacity but not without cost; a fraction of an item was lost from Set 1 for each subsequent item recalled. We present a dual-stage theory in which pointers held in the capacity-limited FoA allow retrieval of chunks from aLTM.

**Supplementary Information:**

The online version contains supplementary material available at 10.3758/s13421-025-01809-7.

Working memory (WM) is the ability to maintain and manipulate information in a heightened state of activation in the brain (cf. Baddeley & Hitch, 1974; Cowan, 2017). WM is limited to only a small number of representations it can simultaneously hold if the material to be remembered comprises a single set of homogeneous stimuli, like verbal lists or visual arrays (Cowan, 2001; Oberauer et al., 2018). Yet humans are capable of incredibly complex thinking and reasoning and often deal with multiple, heterogeneous representations, despite the usual capacity limit of WM. The time is right to aim research at understanding how such a limited capacity can be sufficient for such a range of human capabilities, including, for example, language use, problem-solving, and spatial navigation (Cowan, 2010, 2014).

To address the question of human capability, we ask how people attempt to retain in WM, simultaneously, multiple different sets of items. What limits recall performance in such situations? We devised a novel task that investigates item-based, chunk-based, and similarity-based limitations upon the recall of heterogeneous series of visual stimuli (colors, characters, grid locations, and shapes). The study might be considered akin to the real-world situation of map-reading, in which one must keep track of the colors assigned to different regions, the shapes of those regions, the characters used as symbols for different transit systems, and the locations of stops along the planned route.

Theories differ on how the various nonverbal items in our stimulus set should or should not interfere with one another. Competition between visual items could occur within either a capacity-limited visuospatial sketchpad (Baddeley, 1986; Baddeley et al., 2021) or a general, limited-capacity mechanism like the focus of attention (FoA: Cowan, 2001; Cowan et al., 2024).^1^ In contrast to these theories, if the competition between items depends largely on similarity-based interference (Brown et al., 2007; Nairne, 1990; Oberauer & Lin, 2017), then the total load on WM might be much greater when all items are of the same type (e.g., in our map-reading example, using colors to code both regions and transit systems). The interference from similar representations might be reduced, though, if it is possible to group them as separate ensembles of items (e.g., Frankish, 1985; Parmentier & Maybery, 2008; Son et al., 2020). Below, we examine details of our new, multiple-set paradigm, predictions from the extant literature, and our hypotheses on how WM capacity is constrained when multiple sets of items are to be maintained.

## Design and rationale of the multiple-set paradigm

**Fig. 1:**
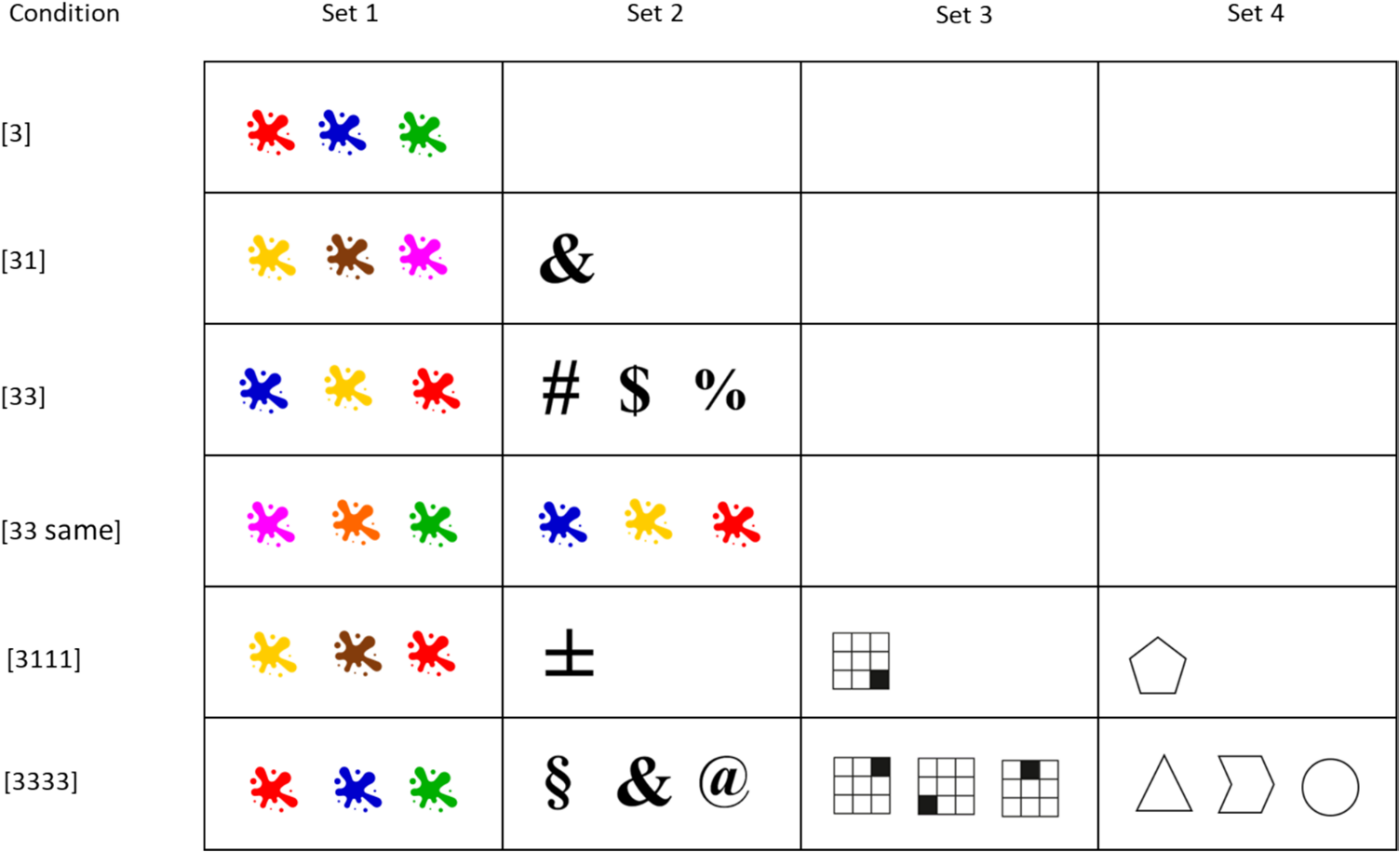
Examples of conditions in the multiple-set paradigm. Items were presented sequentially, and all sets were to be serially recalled from pools of eight possible response choices of each stimulus type. Different sets contained items of different stimulus types except in condition [33 same]. Across the experiment, each stimulus type (colors, characters, locations, and shapes) was presented an equal number of times in each set (Sets 1–4), in an unpredictable order. The number of items in Set 1 was equated across conditions to make comparisons based on the number of subsequent sets/items viable. (Color figure online)

We examined three factors that could limit recall. First is the total load of information in WM—that is, the number of items presented for memorization. Second is the number of sets held in WM; each set included one or three items of a given stimulus type (e.g., one or three shapes). Third is the similarity between sets (e.g., three colors followed by three more colors, versus three colors followed by three shapes). To disentangle the influences of these three factors upon WM capacity, we varied them systematically, always beginning with a set of three items (Set 1) of one stimulus type (Fig. 1). Maintaining the size of Set 1 constant at three items across conditions was key to assess the impact of item-, set-, and similarity-based limitations upon recall of Set 1 and upon the trade-off with other sets.

Each set was presented as a serial list of objects and, following the last set, all sets were to be recalled with the items in the presented order. By assessing the costs to recall of the standard Set 1 of the different experimental conditions, we could determine influences on Set 1. We also could examine the trade-off between recall of Set 1 and other sets. The experimental conditions are illustrated in Fig. 1 and detailed in Table 1.

**Table 1:** Description of the experimental conditions in the multiple-set paradigm

Condition	Set Sizes				Number of Items	Number of sets	Types of stimuli
	Set 1	Set 2	Set 3	Set 4			
[3]	3	—	—	—	3	1	1
[31]	3	1	—	—	4	2	2
[33]	3	3	—	—	6	2	2
[33 same]	3	3	—	—	6	2	1
[3111]	3	1	1	1	6	4	4
[3333]	3	3	3	3	12	4	4

[33] refers to trials with two sets of three objects each, the first set being of a stimulus type and the second set of a different stimulus type, and so on (e.g., condition [3111] could have three colors, one shape, one special character, and one spatial location), except for the [33 same] condition, in which two sets are of the same stimulus type (e.g., three colors and three colors). Across the experiment, each of the four possible stimulus types (colors, characters, locations, and shapes) was presented an equal number of times in each set (Sets 1–4).

Our conditions involved 1) maintaining only Set 1 in WM; 2) maintaining one or more sets in addition to Set 1; 3) maintaining smaller or larger sets concurrently to Set 1; 4) maintaining a second set of items that were either similar or dissimilar to the item type in Set 1. Some conditions were equated in the total number of items and different only in the number of sets (i.e., conditions [33] and [3111]). Others were equated in the number of sets but differed only in the total number of items (i.e., conditions [31] and [33], and [3111] and [3333]). Finally, some conditions were equated in both the number of items and sets and differed only in set similarity (i.e., conditions [33] and [33 same]). By comparing pairs of conditions equated in the total number of items but different number of sets, we can test for a general, feature-independent storage capacity in WM. By comparing conditions with an equal number of sets but different number of items, we can test for a limitation based on the number of sets, assuming that sets are successfully grouped or chunked in WM. Last, the comparison between conditions that differ only in set similarity allows an examination of the role of similarity-based interference with set sizes and number of sets controlled.

## Hypotheses

The multiple-set paradigm allows a comparison of several hypotheses about the limitations upon recall of Set 1. Each should lead to a specific pattern of results that will be described. Table 2 summarizes our hypotheses. Hypotheses 1–3 are alternatives to one another and are mutually exclusive.

**Table 2:** Summary of hypotheses and predictions about recall of Set 1

Hypothesis	Limiting factor of recall			Predicted result pattern
	Number of items	Number of sets	Set similarity	
*H*_*1*_	Yes	No	─	[3] > [31] > [33] = [3111] > [3333]
*H*_*2*_	No	Yes	─	[3] > [31] = [33] > [3111] = [3333]
*H*_*3*_	Yes	Yes	─	[3] > [31] > [33] > [3111] > [3333]
*H*_*4*_	─	─	Yes	[33] > [33 same]

A dash indicates that the specified limiting factor does not apply to the hypothesis listed in Column 1. *H4* is ancillary, and its acceptance is compatible with any outcome derived from Hypotheses 1–3.

- ***H***_***1***_***: Recall of Set 1 is limited by the total number of items in a trial***

We do not endorse this hypothesis but it is what would be expected without chunking. Previous research has shown that about three or four items or well-learned chunks of visual information can be recalled (Awh et al., 2007; Cowan, 2001; Cowan et al., 2012; Luck & Vogel, 1997). If each item must be retained separately, without new chunks being rapidly formed during the trial, the following should be the result:

$$\text{For Set }1, \left[3\right]>\left[31\right]>\left[33\right]=[3111]>[3333]$$

The conditions shown above require retention of 3, 4, 6, 6, and 12 items, respectively, with an increasing total load leading to poorer Set 1 recall.

- ***H***_***2***_***: Recall of Set 1 is limited by the number of sets in a trial***

This outcome would be expected if each set of items is rapidly encoded as newly learned chunk during the trial, so that the capacity limit applies to sets rather than items:

$$\text{For Set }1, \left[3\right]>\left[31\right]=\left[33\right]>\left[3111\right]=[3333].$$

These conditions include 1, 2, 2, 4, and 4 sets, respectively, with an increasing number of sets leading to poorer Set 1 recall.

- ***H***_***3***_***: Recall of Set 1 is limited by both the number of items and sets***

This hypothesis is what would be expected if newly-learned chunks are formed but do not completely, invariably encompass all items in a set. If both the number of items and the number of sets limit recall of Set 1, the result pattern should be:

$$\text{For Set }1, [3]>[31]>[33]>[3111]>[3333]$$

As one moves through the conditions shown here, one finds an increase in the number of items and sets from [3] to [31], the number of items from [31] to [33], the number of sets from [33] to [3111], and the number of items from [3111] to [3333].

A corollary of Hypotheses 1–3 is that, in a capacity-limited system, recall of Set 1 should trade off with recall of other sets at different efficiency ratios, depending on whether item limitations (acceptance of *H1*), set limitations (acceptance of *H2*), or both (acceptance of *H3*) are observed (i.e., something in between *H1* and *H2*). In the *H1* case, the trade-off follows a 1:1 ratio, meaning that each item forgotten in Set 1 corresponds to the recall of one item from another set. In the *H2* case, the trade-off is closer to 1:3, meaning that forgetting one item in Set 1 results in the retrieval of an entire set of three items. In the *H3* case, the trade-off falls between 1:1 and 1:3, reflecting an exchange between individual items in Set 1 and more items in other sets—albeit not complete triads.

- ***H***_***4***_***: Set 1 recall is limited by the feature similarity between sets***

A further, fourth hypothesis can be investigated no matter which of the first three hypotheses is correct. The question here is whether the interference specific to a stimulus type (Nairne, 1990) will result in poorer recall than in a situation in which two sets comprise different stimulus types (e.g., three shapes followed by another three different shapes versus three shapes followed by three colors). If so, for Set 1, [33] > [33 same].

## Theoretical background

The efficient trade-off between Set 1 and other sets require additional reasoning of how item and set-based limitations would apply in WM. Specifically, Rhodes and Cowan (2018) and others (e.g., Cowan, 2019; Cowan et al., 2024) have suggested that series of items can be associated to form new chunks that can be offloaded from a capacity-limited area and held in a temporarily activated portion of long-term memory (aLTM)—activated in that it remains easily retrievable during the trial. This process of offloading reduces the amount of information that must be saved in the capacity-limited part of WM (in Cowan’s terminology, the FoA).

### Pointers and storage efficiency

In some previous descriptions of offloading (e.g., Rhodes & Cowan, 2018), it may have sounded as if the information itself was held in one place—namely, the FoA, and after new associations were formed the information was moved to another place—namely, aLTM outside of the FoA. Here we avoid that implication by using the concept of a *pointer* (cf. Cowan et al., 2011; for a review of the concept applied to cognitive neuroscience, see Awh & Vogel, 2025). The pointer concept comes from computer science, in which a pointer is an object that stores a memory address, at which more detailed information is found. A pointer here refers to a contentless index of an item held in aLTM. By this conception, the capacity-limited contents of the FoA are the number of pointers it holds, with one pointer to each separate entity to be remembered for the task. The entity is a chunk, no matter whether that chunk is a single item or an associated set of items (in the present task, with most of those associations being newly learned).

A multi-item chunk may require a single pointer when it is being maintained or multiple pointers when the chunk is unpacked for recall, with a separate pointer for each item at that point in the trial. Losing track of a single item from Set 1 could allow the space in WM to retain a pointer to another multi-item chunk, to be retrieved later in the trial. Supporting this notion of unpacking, it takes longer to retrieve multi-item chunks than single items (Huang & Awh, 2018).

In physiological terms, a pointer could correspond to the functional connectivity between the intraparietal sulcus, thought to reflect the FoA (see Cowan et al., 2024, for a review), and posterior cortical areas holding the representations in aLTM (Lewis-Peacock et al., 2012; Li et al., 2014). Thus, the pointer does not hold the information about stimuli but shows where to find them for recall. It occupies one slot in the limited capacity of the FoA within the WM system and would appear to operate in the same manner regardless of the modality or code of the stimuli (Majerus et al., 2016).

## Further expectations based on previous studies

In this section we consider two general bases of expectations for the serial recall of Set 1 in our study. First, we consider limitations in the number of items, chunks, or groups that can be remembered. Second, we consider the role of item similarity.

### Limitations based on the number of items, chunks, or groups

There is abundant evidence that, at least for sets of simple, homogeneous visual stimuli, WM is limited in the number of individuated objects it can hold—about three or four items (Adam et al., 2017; Cowan et al., 2011; Luck & Vogel, 1997; Ngiam et al., 2024). For example, adult participants can hold only about three to four color–location associations in WM (Cowan et al., 2011). This capacity limit is reflected in neural measures such as the contralateral delay activity (CDA; for a review, see Luria et al., 2016). The CDA appears to reflect a content-independent load signal, showing similar patterns for different feature types such as color and orientation (Jones et al., 2024). Moreover, neural signatures of WM load generalize across memory conditions that vary in both the type and number of features per item, suggesting a flexible but object-based storage system (Balaban et al., 2019; Thyer et al., 2022; Woodman & Vogel, 2008). Similarly, Rajsic et al. (2019) demonstrated that CDA amplitude scales with set size across various stimulus types—including colored squares, letters, and words—indicating a general mechanism for object-based storage in visual WM. Most of this evidence, however, has been gathered in studies that did not use diverse, multiple groups of visual items, but rather required memory for a homogeneous (drawn from one stimulus type) group of items presented either as series or concurrent array (for an exception with two types of items, see Markov et al., 2019).

In studies that investigated limitations on the number of visual chunks or groups, the traditional approach has been to use one-dimensional stimuli (e.g., all items are color patches, or all black shapes). When items in a simultaneous array are grouped by the Gestalt principles of spatial proximity and connectedness, WM capacity is optimized (Woodman et al., 2003; Xu, 2006). Grouping based on similarity also improves WM capacity for items near one another in the array (Brady et al., 2011; Peterson & Berryhill, 2013).

Many studies on verbal WM capacity examine situations in which items can be temporally segmented into smaller clusters. Grouped items are typically easier to remember than if there were no grouping, for two reasons. First, the items may form multi-item, known chunks (Cowan et al., 2012; Miller, 1956), like the acronyms IBM (for International Business Machines) and CIA (for Central Intelligence Agency). In that case, about three or four chunks typically can be remembered (Cowan et al., 2004, 2012). Second, and of more direct relevance to the present work, grouping may allow new associations between items or between each item and its context, even in the absence of known chunks. These newly formed associations are beneficial to WM capacity even though they are generally not as effective as well-learned, known chunks. Examples are groups of spoken digits presented sequentially and divided by silent periods or some other grouping marker (Frankish, 1985; Parmentier & Maybery, 2008; Ryan, 1969a, 1969b; Spurgeon et al., 2015).

For visual presentation of verbal materials, the absence of physical grouping cues, like silent periods or tones, does not prevent participants from imposing their own mental grouping (Cowan & Hardman, 2021; Cowan et al., 2002). There is no set limit for how many groups can be perfectly remembered, but it seems in keeping with extant findings that the capacity limit of three or four items applies also to how many groups can be at least partially remembered (e.g., the number of lists of unrelated sentences recalled with about 80% of the words in each sentence: Gilchrist et al., 2008). Our work extends temporal grouping methods to the visual domain, with sequences of visual objects instead of letters and digits. As an example of study with visual sequences, Forsberg et al. (2025) recently examined serial position effects for lists of up to six visual objects in WM (and transfer to LTM), but did not examine grouping effects or heterogeneity of the materials as in the present study.

There has been insufficient attention paid to the possibility that there are dual constraints on WM capacity: constraints in terms of (1) the total number of items and (2) the number of groups to be remembered. There are several ways that both constraints could occur. Chen and Cowan (2005) found that item-based and chunk-based limitations operate in different circumstances in verbal recall, with a general item limit governing serial recall with strict scoring, presumably because of rehearsal time, and a chunk limit governing serial recall with lax scoring or free recall, presumably because of a capacity limit. In the former case, the authors observed similar performance regardless of lists comprising eight singletons or four learned word pairs, suggesting a limit of eight words. In the latter case, they observed similar performance regardless of lists comprising six learned word pairs or six learned singletons, suggesting a limit of six chunks. In a later study, Chen and Cowan (2009) showed that, under articulatory suppression, capacity estimates were the same for lists comprising singletons and well-learned word pairs. When covert rehearsal was suppressed, participants consistently recalled about three chunks, regardless of list length or chunk size, supporting the idea of a central, chunk-based capacity limit in WM when rehearsal is prevented. Importantly, both studies suggest that single words and well-learned word pairs function in the same way as individual items in WM. A later study (Cowan et al., 2012) extended the capacity concept to familiar triads such as *leather brief case*, showing with a mathematical model that capacity limited to about three or four chunks generally holds across various chunk sizes. Content-free pointers that index various loads of information are a viable mechanism to describe this central limitation irrespective of chunk size (Awh & Vogel, 2025).

Here, unlike Chen and Cowan (2005, 2009), we presented nonverbal items that were expected to be difficult to rehearse verbally, and that allowed us to present sets of items from different stimulus types on the same trial to reduce interference effects. No chunks should exist in long-term memory for our materials; thus, our experiment taps into rapid chunk formation at encoding. Participants could rapidly attempt to chunk items within a set of the same stimulus type (e.g., a chunk containing three associated colors), thereby assigning the whole set to a single pointer and optimizing storage. However, if the chunking of items into sets is incomplete (e.g., in a set of three colors, two colors are chunked and a third color is coded as a singleton), then there should still be effects of the number of items presented, and not just the number of sets that theoretically could be converted into chunks.

### The role of feature similarity in grouping effects

In experimental situations enabling grouping, like in the studies by Frankish (1985), cited above, the similarity of items in a trial might hurt recall because of confusions between items and even between groups. For example, consider a trial in which items of the same type (e.g., digits, D) are segmented into groups of three (e.g., D_1_D_2_D_3_─D_4_D_5_D_6_─D_7_D_8_D_9_). Confusions about the serial position of the digits might arise either at the trial level (e.g., reporting an item that was not presented in the current trial, but in a previous trial – proactive interference); the group level (e.g., swapping items between groups, but maintaining their position within a group, like reporting D_7_D_5_D_6_─D_4_D_8_D_9_ instead of D_4_D_5_D_6_─D_7_D_8_D_9_); or the position level (e.g., swapping serial positions within a group, like reporting the second group as D_5_D_4_D_6_ instead of D_4_D_5_D_6_). Processes like these were examined by Lee and Estes (1981) by using series of digits and letters. When items are homogeneous across all groups in a trial, errors at the group level are likely to occur due to confusion on the identity of items in different groups, i.e., a matter of group membership (cf. Experiment 3 of Lee & Estes).

Cowan and Hardman (2021) found that an extreme case of similarity between items—namely, item repetition—can be either helpful or deleterious for recall. They presented participants with lists of nine digits with various patterns of item repetitions, and the lists could either have a grouped or ungrouped spatial structure of concurrent visual presentation (e.g., D_1_D_2_D_3_─D_4_D_5_D_6_ ─D_7_D_8_D_9_, versus D_1_D_2_D_3_D_4_D_5_D_6_D_7_D_8_D_9_, respectively). Note that participants were free to group stimuli according to any structure they wished in the latter condition. Item repetition was helpful when it reinforced a grouping structure that was given to participants (e.g., repetitions within the same group, “777–832–564,” or at comparable serial positions within groups, “742–795–768”). Item repetition was also helpful when participants were free to impose their own grouping structure. However, item repetition was harmful when it conflicted with the given grouping structure, especially when they occurred in different serial positions within different groups (e.g., “742-975–687”).

Considering the research of Lee and Estes (1981) and Cowan and Hardman (2021) together, we expected that similarity effects can interact with grouping in ways that complicate the processes taking place for grouped stimuli. To simplify the situation, most of our trials with multiple sets include three items of one stimulus type (e.g., three characters) forming a critical first set and each subsequent set is drawn from a different stimulus type (e.g., a critical set of three characters, followed by three colors, three grid locations, and three shapes). This kind of arrangement of stimuli is rare in experimentation but common in daily life. For example, a server at a restaurant may have to try to remember three appetizers, three types of soup, three main courses, and three desserts for a table. By allowing categorical similarity within but not between groups, we try to ensure that similarity-based synergies or confusions between groups do not contribute to the difficulty of the task, allowing us to assess the contribution of the independent loads imposed by those groups to WM capacity.

In one exception to the structure of groups formed of different stimulus types, our condition [33 same] includes trials with two sets of items drawn from the same stimulus type in order to assess effects of intergroup similarity. Our expectation was that the similarity between groups would be detrimental to performance overall because of group membership confusion. However, we were unsure if group membership confusion would be important for our visual stimuli, inasmuch as confusion theoretically could be an unintended consequence of verbal rehearsal applied to letters and digits in previous studies (Cowan & Hardman, 2021; Lee & Estes, 1981).

To our knowledge, no study has extensively tested grouping effects in the temporal domain with sets of heterogeneous stimulus types as in this study. In perhaps the closest precursor, Lee and Estes (1981, Experiment 2) presented 12-item lists for serial recall, each list including an initial group of four letters, a group of four digits, and final group of four letters. There would still be the possibility of letters from the first and final groups in the list being confused with one another in terms of group membership. Using multiple heterogeneous item types, and doing so in the visual domain, can give a more optimal estimate of WM capacity without confusions of group membership or verbal rehearsal processes.

Similarity between sets might modulate the dynamics of grouping. The joint constraints of item limitations, set limitations, and inter-set similarity on WM capacity constitute the question of the current study. When multiple sets of items are to be maintained in WM, how do memorized sets of different types compete for a limited capacity? We set up an experimental paradigm in which this question could be addressed in great detail for the first-presented, first-recalled set and in lesser detail for the varying, remaining sets.

## Experiment 1

### Method

#### Participants

Sixty-one participants between 18 and 30 years old (25 women, 33 men, three nonbinary, *M*_age_ = 25.5 years, *SD* = 3.1) took part in our study via the online platform Prolific; no one was excluded from our analyses. The inclusion criteria were 1) people living in the U.S., and 2) English speakers. Participants filled out a questionnaire on their demographic information (sex, race, ethnicity, years of education, and languages spoken) and on medical information (visual and hearing acuity, presence cognitive impairments, and medication intake) prior to taking part in the experiment. Participants could choose not to disclose information by answering “Prefer not to say” to all questions in the questionnaire. Response options to the question on sex were "Male", "Female", "Other", and "Prefer not to say". Response options to the question on race were “American Indian/Alaska Native”, “Asian”, “Black or African American”, “More than one race”, “Native Hawaiian or other Pacific Islander”, “White or European”, “Unknown”, “Other”, and “Prefer not to say”. Response options to the question on ethnicity were “Hispanic or Latino”, “Not Hispanic or Latino”, “Other”, and “Prefer not to say”. These questions were followed by the open-ended question “If you selected ‘Other’, you may provide additional information if desired.” All participants gave written consent prior and were given monetary compensation for taking part in the study. All procedures complied with ethical standards and were approved by the Institutional Review Board at the University of Missouri (protocol #99-04-095).

#### Sample size determination

Because our design is novel and there are no effect sizes reported in the literature, we started by running a sample size estimation for a frequentist repeated-measures analysis of variance (ANOVA) on G*Power. We used standard values in the field as input: Cohen’s *f* = 0.25 (medium effect size), alpha = 0.05, power = 0.95. This yielded 28 as the minimum sample size required. Because Bayesian tests require larger sample sizes to reject the null hypothesis than to accept the alternative hypothesis, we decided on the basis of an available, still somewhat rudimentary counterpart to power analysis for Bayesian designs (Schönbrodt & Stefan, 2019) to more than double the sample size, to 60 participants per experiment.

#### Apparatus

The experiment was programmed and implemented in Psytoolkit (Version 3.4.4; Stoet, 2010, 2017) and the experimental sessions were online. Participants were required to use a computer while taking part in the task.

#### Stimuli

The pool of stimuli consisted of sets of colors, shapes, spatial locations, or special characters (Fig. 1). The colors were red (RGB: 255, 0, 0), blue (RGB: 0, 0, 255), green (RGB: 0, 175, 0), pink (RGB: 255, 0, 255), purple (RGB: 153, 0, 255), yellow (RGB: 255, 204, 0), orange (RGB: 255, 102, 0), brown (RGB: 131, 60, 0), presented in the format of an inkblot shape. The shapes were a circle, a square, a triangle, a diamond, a pentagon, a trapezoid, a semicircle, and an arrow. The spatial locations were defined by one filled cell among nine cells in a 3 × 3 grid. The special characters were a hash (#), an ampersand (&), an at sign (@), a percent sign (%), a dollar sign ($), a pilcrow (¶), a plus–minus sign (±), and a section sign (§), set up in the font Times New Roman and size 64. Stimuli covered about 17% of the viewing area of the screen. Our stimuli and tasks are available on OSF (https://osf.io/2abxs).

#### Design

**Fig. 2:**
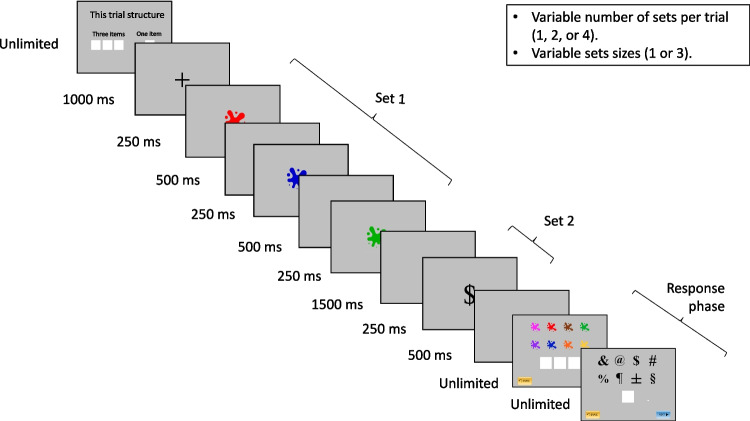
Sequence of events in a trial of Experiment 1. The example depicts a trial in condition [31], with one set of three colors and one set of one special character. Note that the two sets are separated by a longer ISI of 1,500 ms. See Table 1 for a list of conditions and Fig. 1 for graphic examples of conditions. In the response phase, participants chose their answers from a multiple-choice set to fill in the response slots (white squares) at the bottom of the screen, by clicking on a response option. The stimuli are not to scale. (Color figure online)

Experiment 1 implemented a multiple-choice serial recall task. Participants were presented with sequences of colors, shapes, spatial locations, and special characters and asked to reconstruct the order of presentation of the stimuli on every trial. We manipulated the number of sets and the set sizes presented in each trial (see Table 1 for a summary of the experimental conditions). The number of sets corresponds to the number of groups of one or more items temporally distinct from other items, separated by a longer interval than the within-set interval. The number of sets could be one, two, or four and each set could contain one or three items. Among the combinations chosen for the experiment, the total number of items presented in a trial could be three, four, six, or 12. No two sets included items of the same stimulus type except in the [33 same] condition. Participants were instructed to memorize all items presented during the trial, and each item was to be serially recalled from a multiple-choice display containing the eight possible stimuli from a given stimulus type within a set. Items were tested in a set-by-set fashion (i.e., Set 1 was tested, then Set 2, so on, depending on the specific experimental condition). Figure 2 represents the complete sequence of events in a trial, using condition [31] as an example.

#### Procedure

Each trial was preceded by the presentation of a schematic visual representation of the trial structure in terms of the number of sets and set sizes in the specific condition (see Fig. 1 for an example). In this visual representation, the words *“This trial structure:”* were printed at the top of the screen and blank, horizontally aligned squares representing each item were presented at the center of the screen, with the words “*one item*” or “*three items*” appearing above the representation of each set, depending on the set size. Participants were given free time to observe this trial structure screen, and then pressed the spacebar to initiate the trial. Each trial started with a fixation cross for 1,000 ms, followed by the central presentation of items for 250 ms, interspaced by a blank interstimulus interval (ISI) of 500 ms. In conditions with more than one set, an ISI of 1,500 ms separated the different sets. At the end of the trial, participants reconstructed the order of presentation of items in each set by clicking on response options on multiple-choice displays. The configuration of the multiple-choice display was fixed throughout the experiment (i.e., choice items were always presented in the same location). The response screens showed the eight possible items from a given stimulus type, and white boxes representing serial positions (in a left-to-right orientation) within a set. Upon a mouse click, the chosen item became unavailable and appeared in the respective white box. Sets were tested in the same order of presentation (i.e., participants reconstructed the order of items in Set 1, then in Set 2, and so on). Participants were allowed to change their latest response, but they could not return to previous responses after moving to the next item of choice. Advancing to the next set was only possible after completing all responses for the current set. There was no time limit for responding.

Each experimental condition had 12 trials, for a total of 72 trials in the experiment. Each of the four possible stimulus types (colors, characters, locations, and shapes) were presented the same number of times in each set (Set 1, Set 2, Set 3, and Set 4). On each trial, items were randomly drawn from the pool of eight stimuli belonging to a given stimulus type, with no repetition within the same set. Conditions were manipulated between trials that were randomly intermixed. The progression of the trials was self-paced, and no feedback was given during test trials. Test trials were preceded by a familiarization phase in which participants were presented with the stimuli pool, instructed on how to use the response screens, and did 12 practice trials in which response feedback was given. The average testing time was about 45 min.

#### Data Analysis

The key analysis involved *k*_Si_, an estimate of the number of items known from any one set, in which *Si* designates the specified set. It was estimated from the proportion of items correct on that set, separately for each participant in each condition after correcting for guessing. We adopted a strict, position scoring to calculate the proportion of items correct and to determine *k*_Si._

It was assumed each item in a set was known and recalled with *p*(known) = *k*_Si_*/SS*_i_, in which *SS*_i_ represents the set size of that given set—this value could be one or three items. For example, if two of three items in Set 1 were known, then the probability of knowing any one particular item in that set would be *p*(known) = 2/3. Among the items in a set that are not known, each one is randomly guessed from among 8 − *k*_Si_ choices (i.e., the total number of response options minus the number of known items), inasmuch as the values of known items are unavailable for these choices for guessing. This is because choices are made without replacement in the response screen and items were never repeated within a set. The guessing process governs *p*(not known) = 1 − (*k*_Si_/*SS*_i_) of the choices. Therefore, the proportion correct on an item in any given set should be decomposed into knowledge plus guessing processes, as shown in Appendix A.

We compared the number of items recalled from Set 1 and from the other sets by using Bayesian one-way repeated-measures ANOVAs with condition (as presented in Table 1) as a within-participant factor, and by running Bayesian *post hoc* comparisons to contrast all the pairs of conditions (JASP Team, 2023). Further examining the data, we also report how recall in the other sets varies as a function of recall in Set 1. We used frequentist ANOVAs for descriptive purposes and Bayesian ANOVAs for inferential purposes. Q-Q plots showing a normal distribution of residuals of participant means are available in the supplementary materials.

#### Transparency and openness

The study materials, program code, and dataset are available on OSF (https://osf.io/2abxs/). This study and analysis plan were not preregistered.

### Results

#### Number of items recalled from Set 1

**Fig. 3:**
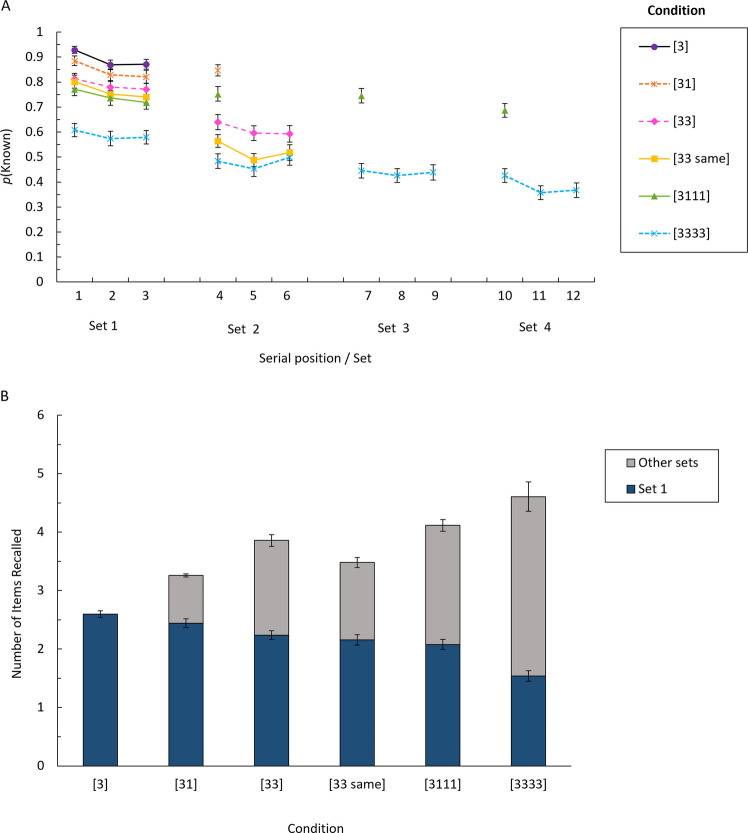
Performance per experimental condition of Experiment 1. **A** Probability of knowing an item (*p*(known)) in each serial position, per experimental condition of Experiment 1. **B** Number of items recalled from Set 1 and from other sets, collapsed across serial positions; the stacked columns represent the total number of items recalled across sets in a trial. Vertical bars represent standard errors. (Color figure online)

Performance in Experiment 1 is shown in Figure 3 and detailed in Table 3. The one-way ANOVA comparing the number of items recalled from Set 1 resulted in a Bayes factor (BF_10_) of 1.85×10^44^ for the inclusion of the effect of condition (JASP Team, 2023), and a very large effect size (η_p_^2^ = 0.53). The post hoc comparisons showed BF_10_ > 100,000 for most comparisons, with smaller but still reliable differences for condition [3] (*M* = 2.60, *SD* = 0.45) versus condition [31] (*M* = 2.44, *SD* = 0.59), BF_10_ = 22.28, and for condition [33] (*M* = 2.24, *SD* = 0.58) versus condition [3111] (*M* = 2.08, *SD* = 0.69), BF_10_ = 6.54. This last comparison establishes the importance of the number of distinct sets to be held in WM when the total number of items is constant across the compared conditions. All these contrasts are consistent with *H*_*3*_, in which [3] > [31] > [33] > [3111] > [3333]. This hypothesis states that both the number of items and the number of sets limit storage capacity in WM.

**Table 3:** Number of items recalled from Set 1 and from other sets, per condition of Experiment 1

Condition	Number of items recalled in Set 1	Number of items recalled in other sets
[3]	2.60 (0.06) [2.48, 2.71]	-
[31]	2.44 (0.08) [2.29, 2.59]	0.82 (0.03) [0.77, 0.87]
[33]	2.24 (0.08) [2.09, 2.39]	1.62 (0.10) [1.42, 1.82]
[33 same]	2.16 (0.09) [1.98, 2.33]	1.32 (0.09) [1.15, 1.49]
[3111]	2.08 (0.09) [1.90, 2.26]	2.04 (0.10) [1.84, 2.24]
[3333]	1.54 (0.09) [1.37, 1.71]	3.07 (0.25) [2.56, 3.57]

Values between parentheses represent the standard errors and values between brackets represent 95% credible intervals.

The only nonreliable differences were between condition [33 same] (*M* = 2.16, *SD* = 0.68) and condition [33] (*M* = 2.24, *SD* = 0.58), BF_10_ = .45, and between condition [33 same] and condition [3111] (*M* = 2.08, *SD* = 0.69), BF_10_ = .36. These indeterminate results suggest that the similarity between sets made little difference for the number of items recalled from Set 1, against *H*_4_. In sum, the evidence supports *H*_3_, stating a role for both the number of items and the number of sets, and therefore supports a dual-level mechanism of maintenance of items in WM (Cowan, 2019; Rhodes & Cowan, 2018), with no clear role for similarity across sets. It is quite possible that there is a role of similarity within a set (e.g., Oberauer & Lin, 2017) but that the dissimilarity afforded by set membership (in this task, defined by longer inter-set intervals) is enough to prevent feature similarity factors from being important in determining recall performance for Set 1.

#### Number of items recalled from other sets

When considering the number of items recalled in the other sets (Fig. 3C and Table 3), we also found decisive evidence for an effect of the experimental condition (BF = 3.50×10^34^) and a very large effect size (η_p_^2^ = 0.52). Post hoc comparisons showed compelling evidence that the mean number of items recalled was different between each tested pair, with all BFs > 3,000. Interestingly, we observed that memory for the other sets was higher in condition [33] (*M* = 1.62, *SD* = 0.78) than in [33 same] (*M* = 1.32, *SD* = 0.67), which share the same structure but differ in terms of the similarity between the sets. Moreover, memory for the other sets was also higher in [3111] (*M* = 2.04, *SD* = 0.10) than in both [33] and [33 same]. A possible explanation was that pauses between single-item sets in [3111] could have also played a role, by providing each item with a more distinctive temporal context than in the second set of condition [33]. In advance of our Experiment 3 results, the same result was observed when sets were presented without interspaced pauses (cf. Experiment 3), therefore ruling out the interpretation based on temporal distinctiveness. The clear conclusion is that similarity matters when a second set is recalled, with performance levels being lower in the second set with increased levels of inter-set similarity. This proactive interference could occur either during encoding of the second set or during recall due to having previously recalled Set 1. 

#### Trade-off between recall of Set 1 and other sets

In a capacity-limited system, adding sets after Set 1 is expected to reduce recall from that set, while still allowing some information from additional sets to be recalled, as WM attempts to manage all of the stimuli. In this multi-set scenario, a trade-off between recall in Set 1 and other sets is expected. To assess the nature of this trade-off, we performed a linear regression analysis between the number of recalled items in Set 1 and those in the other sets. We observed a negative linear relationship, according to which, each loss of one item from Set 1 was accompanied by an increment of 1.77 item recalled from the other sets—thus suggesting an efficient trade-off. The relationship between the number of items recalled from Set 1, other sets, and the total number of items recalled across sets is represented in Fig. 3B, and the regression between recall from Set 1 and other sets is shown in Fig. 5C.

### Discussion

Experiment 1 aimed at investigating the constraints to WM capacity while concurrently maintaining more than one set of visual items for recall. We designed a paradigm in which we varied the number of items presented in a trial, the number of sets, and the similarity between the sets, which allowed us to test multiple hypotheses. The novel hypothesis was that performance in a critical Set 1 would depend on the number of additional sets presented in a trial, but not on their respective set sizes (*H*_2_), reflecting a chunking process based on set membership.

We observed a strong effect of the experimental conditions upon the number of items recalled from Set 1, and decisive evidence (BFs > 3) for differences between most of the tested paired conditions. First, the full pattern of performance in Set 1 has been predicted by *H*_3_, which supports the idea that both the total number of items and the number of sets presented in a trial limit WM capacity in experimental situations involving the maintenance of multiple sets of visual items. Second, the observation that Set 1 recall in [33] was superior (albeit only a little) to [3111] ruled out the hypothesis of a purely item-based, general limit in WM (*H*_1_), given that both conditions have the same number of items. Moreover, the observation that recall was different between pairs of conditions sharing the same number of sets, but with different set sizes ([31] versus [33], and [3111] versus [3333]), ruled out the hypothesis of a limitation based exclusively on the number of sets kept in WM (*H*_2_)*.* Hence, the results strongly support *H*_3*,*_ a limitation in both the number of items and the number of sets that can be recalled. Third, the observation that Set 1 recall in condition [33 same] was not reliably different from conditions [33] and [3111] did not support *H*_4,_ stating a limitation based on the similarity between the sets*.* Last, we found an efficient trade-off between the information recalled from other sets and from Set 1, with 2.77 items recalled from other sets for every item lost from Set 1 (Fig. 3B and Fig. 5C).

## Experiment 2

One alternative interpretation of the results in Experiment 1 is that performance in Set 1 could be governed by the number of interfering events between input and output. Interfering events are conceptualized as the encoding of subsequent items after a particular item is encoded, and prior responses before that item is recalled. According to this account, more events can interfere with a given representation in WM before it is to be recalled, hindering recall performance (Bartsch & Oberauer, 2023).

Such an interpretation is applicable to our Experiment 1 because we tested lists in forward order, therefore the number of events between the encoding (input) and retrieval (output) of an item were fully confounded with the total number of items presented for memorization. For example, if the list ABCD is presented and tested in this order, the input-output distance is 3 for all tested items (i.e., there are three interfering events between the encoding and the recall of an item). For the critical item A, 3 is also the number of additional representations held in WM at recall (i.e., items BCD, which will still be later used for response). If, however, the list was tested in backward order, the input-output distances for items A, B, C, and D would be 6, 4, 2, and 0, respectively. Across recall orders, the number of items to be recalled can be dissociated from the amount of interference due to input-output distance.

In Experiment 2, we held the number of items constant at 6 and disentangled the number of interfering events from the size and number of sets by manipulating not only the number of sets, but also the order of presentation and the order of test of these sets. We selected conditions [33], [33 same], and [3111], all of which have six total items. By manipulating the order of presentation and test in these conditions, we could widely vary the input-output distances of the critical set for several of our hypotheses about Set 1 recall. Now, the interference from other sets could be made very small, if the critical set was presented last and tested first (0 interfering events); it could be made very large, if the critical set was presented first but tested last (6 intervening events, with both input and output interference); or it could be intermediate, if it was presented and tested first (3 interfering events, only input interference upon the critical set) or presented and tested last (3 interfering events, only output interference). If the number of interfering events accounts for all of the results in Experiment 1, our capacity-based interpretation would be called into question. Also, the manipulation of the order of presentation of test of the critical set also allows us to pinpoint the locus of interference at encoding (input interference) and/or retrieval (output interference).

To avoid confusion with the order of presentation and test of sets in Experiment 2, we will henceforth call them Sets A, B, C and D instead of Sets 1–4, as shown in Table 4. Set A (comprising items A_1_, A_2_, A_3_, and A_4_) is always the critical set for comparison with Set 1 in Experiment 1. In short input-output distance conditions, the critical Set A was presented last but tested first (*p*Last-*t*First). For example, in condition [3111], the *p*Last-*t*First order of presentation was BCDA_1_A_2_A_3_ and the order of test was A_1_A_2_A_3_BCD. Note that we did not vary the order of presentation and test by making it in a classical forward and backward order, but rather just inverted the position of the critical Set A so that it was the first and/or the last to be presented and/or tested. This is because items within a set were always to be recalled in the forward order, so that we could avoid the extra burden in WM associated with the operation of backward recall. Moreover, the option for just reversing the position of the critical Set A allows us to keep the input-output distance of the other sets constant (e.g., in the exemplified BCDA_1_A_2_A_3_ – A_1_A_2_A_3_BCD above, the output-input distance is the same for the sets B, C, and D).

**Table 4:** Description of the conditions in Experiment 2

Condition	Set size (A)	Set size (B)	Set size (C)	Set size (D)	In-out distance (Set A)	Locus of interference	Trial description (encoding | test)
[3111]_*p*First-*t*First	3	1	1	1	3	Input	A_1_A_2_A_3_BCD | A_1_A_2_A_3_BCD
[3111]_*p*First-*t*Last	3	1	1	1	6	Input and output	A_1_A_2_A_3_BCD | BCDA_1_A_2_A_3_
[3111]_*p*Last-*t*First	3	1	1	1	0	__	BCDA_1_A_2_A_3 _| A_1_A_2_A_3_BCD
[3111]_*p*Last-*t*Last	3	1	1	1	3	Output	BCDA_1_A_2_A_3 _| BCDA_1_A_2_A_3_
[33]_*p*First-*t*First	3	3	-	-	3	Input	A_1_A_2_A_3_B_1_B_2_B_3_ | A_1_A_2_A_3_B_1_B_2_B_3_
[33]_*p*First-*t*Last	3	3	-	-	6	Input and output	A_1_A_2_A_3_B_1_B_2_B_3_ | B_1_B_2_B_3_A_1_A_2_A_3_
[33]_*p*Last-*t*First	3	3	-	-	0	__	B_1_B_2_B_3_A_1_A_2_A_3_| A_1_A_2_A_3_B_1_B_2_B_3_
[33]_*p*Last-*t*Last	3	3	-	-	3	Output	B_1_B_2_B_3_A_1_A_2_A_3_| B_1_B_2_B_3_A_1_A_2_A_3_
[33 same]_*p*First-*t*First	3	3	-	-	3	Input	A_1_A_2_A_3_B_1_B_2_B_3_ | A_1_A_2_A_3_B_1_B_2_B_3_
[33 same]_*p*First-*t*Last	3	3	-	-	6	Input and output	A_1_A_2_A_3_B_1_B_2_B_3_ | B_1_B_2_B_3_A_1_A_2_A_3_
[33 same]_*p*Last-*t*First	3	3	-	-	0	__	B_1_B_2_B_3_A_1_A_2_A_3_ | A_1_A_2_A_3_B_1_B_2_B_3_
[33 same]_*p*Last-*t*Last	3	3	-	-	3	Output	B_1_B_2_B_3_A_1_A_2_A_3_ | B_1_B_2_B_3_A_1_A_2_A_3_

In each condition name, the extensions *p*First-*t*First, *p*First-*t*Last, and so forth, indicate the order of presentation and test of the critical Set A, respectively (*p*First = presented first, *p*Last = presented last, *t*First = tested first, *t*Last = tested last). In the column “Trial description,” the vertical bars separate the presentation phase from the response phase, and the subscribed numbers indicate items belonging to the same feature set (e.g., A_1_A_2_A_3_ indicate three items of the same stimulus type in Set A). In conditions [33 same], both Sets A and B had the same stimulus type. The presentation of each possible stimulus type was equalized across the sets.

Table 4 details the order of events in a trial in each condition of Experiment 2 and indicates the input-output distance for the critical Set A. Note that, from the participant’s perspective, the order of events taking place in the trial for conditions [33] and [33same] is essentially the same for the order conditions *p*First-*t*First compared to *p*Last-*t*Last, and *p*First-*t*Last compared to *p*Last-*t*First. However, these order conditions are inherently different in terms of the order of encoding and test of the critical set A, so that the locus of input and output interference differs between them.

If the governing factor of memory capacity in Experiment 1 was the amount of interference between the items in the memoranda, then we should observe better memory for the critical Set A in conditions with short input-output distances. On the other hand, if item interference is not an essential factor, then we should not observe an effect of the input-output distances.

A secondary purpose of Experiment 2 was to take a closer look at the role of similarity between sets. In Experiment 1, similarity did not have a clear impact on recall of the critical Set 1, but it affected recall of the subsequent three items in a trial. In that experiment, the difference between Sets 1 and 2 was that Set 1 was tested after three intervening input events (i.e., the presentation of the other sets), whereas the second set was tested after three intervening output events (i.e., the responses for the critical Set 1). Perhaps only output events increase the role of similarity upon capacity. If this is the case, then the similarity effect should be larger in both of the (output last) conditions than in the (output first) conditions, but should not differ between the (input first) and (input last) conditions.

### Method

#### Participants

Sixty participants (*M*_age_ = 25.76 years, *SD* = 3.02 years, 21 women, 35 men, four nonbinary or nondeclared) took part in Experiment 2 on Prolific, and none of them had taken part in Experiment 1. All participants were included in the analysis.

#### Stimuli

All stimuli and time parameters were identical to those used in Experiment 1.

#### Design and procedure

Experiment 2 had 12 conditions resulting from the combination of three possible set configurations ([3111], [33], and [33 same]), two possible orders of presentation of the critical Set A (presented first, presented last), and two possible orders of test of Set A (tested first, tested last). Table 4 details the experimental conditions of Experiment 2. The experiment totaled 12 practice trials (one of each condition) and 96 trials (eight per condition), and the conditions were randomized between trials. The presentation of the four possible stimulus types (colors, locations, shapes, and special characters) was equalized across sets A–D by using a Latin square. The average testing time was about 1 hour.

Participants were instructed to memorize the sequences of items and told that sets could be tested in any order. As in Experiment 1, participants were informed about the order of presentation of the sets prior to each trial (see Fig. 2, first screen “This trial structure”), but not about the order of the test. In the response phase, a miniature version of the trial structure was shown at the bottom part of the response screen with an arrow indicating which set was currently being tested. Moreover, the miniature slots in the scheme were filled in black as the responses were submitted, providing participants with a progress report (but not feedback) of their responses. Figure S1 in the supplementary material represents all possible orders of presentation and test in Experiment 2.

#### Data analysis

The number of items recalled was calculated and corrected for guessing in the same manner as in Experiment 1. For the critical Set A, we compared the number of recalled items across conditions by using a Bayesian three-way repeated-measures ANOVA with the factors set condition ([3111], [33], [33 same]), the order of presentation of Set A (presented first, presented last), and the order of test of Set A (tested first, tested last). The most important analysis was the observation of the number of items recalled for all conditions as a function of the input-output distance.

### Results

#### Number of items recalled from Set 1

**Fig. 4:**
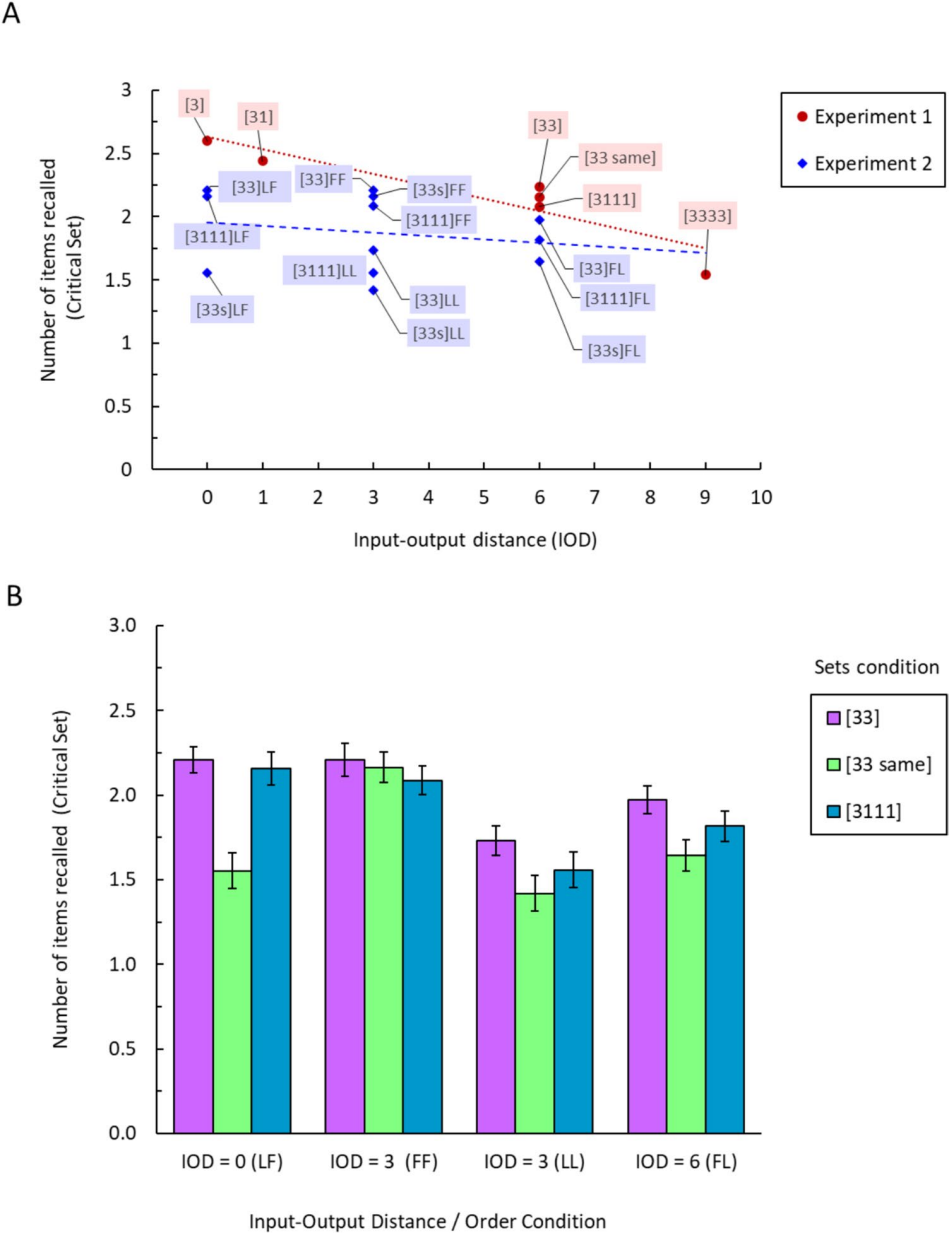
Number of items recalled from the critical set in Experiments 1 and 2 as function of input-output distance (**A**), and in each condition of Experiment 2 (**B**). Panel **A** represents the mean number of items recalled from the critical set of Experiments 1 and 2. The dashed lines represent the linear trend between the number of items recalled and the input-output distances. Panel **B** represents the mean number of items recalled from the critical Set A of Experiment 2, in each order condition. Vertical bars represent the standard errors. FF = presented first, tested first; FL = presented first, tested last; LF = presented last, tested first; LL = presented last, tested last. IOD = input-output distance. (Color figure online)

To examine the alternative interpretation of the results based on the number of interfering events, we plotted the number of items recalled from the critical set as a function of the input-output distance in Experiments 1 and 2 (Fig. 4A). The fit is excellent in Experiment 1 but not in Experiment 2. The number of items recalled from the critical set was not drastically impaired in conditions with more interfering events between presentation and test, and it did not even improve in conditions with zero interfering events for that set. There does seem to be an effect of output interference from response production, given the disadvantage for sets tested last. (This was corroborated by the very strong effect of the test order in the model.) However, this influence of output interference cannot explain the effects of the number of items that we observed in Experiment 1, in which the critical set was always tested first (hence with no prior output interference).

Figure 4B illustrates performance in the critical Set A in all conditions of Experiment 2. The mean number of items recalled from the critical set replicated almost exactly the results observed for Set 1 in the comparable conditions in Experiment 1 (i.e., conditions [3111], [33], and [33 same] of Experiment 1 compared with conditions *p*First-*t*First in Experiment 2). This replication was confirmed by a 3 × 2 ANOVA comparing these conditions between the two experiments, with no evidence for an experiment effect (BF_incl_ = 0.32). Moreover, the relationship observed between the number of items recalled from the critical set and the other sets in the comparable conditions of Experiment 1 was also replicated closely in Experiment 2 (cf. Table S1 in the supplementary materials for the full descriptives, and Fig. S2A, for a visual representation of the comparable conditions).

Comparisons across matched models showed reliable evidence for the inclusion of the sets condition, BF_incl_ = 1.96×10^10^ (η_p_^2^ = 0.40); the order of test, BF_incl_ = 2.94×10^11^ (η_p_^2^ = 0.65); the order of presentation, BF_incl_ = 408.69 (η_p_^2^ = 0.25); the interaction between set condition and presentation order, BF_incl_ = 131.67 (η_p_^2^ = 0.14); and the three-way interaction, BF_incl_ = 1.36×10^6^ (η_p_^2^ = 0.19). Evidence was against the inclusion of the other two interactions, BF_incl_ < .3 in both cases. According to the model, memory for the critical set was overall hindered when it was tested last, and slightly worse when it was also presented last. The effect of test order is clearly seen in conditions [33] and [3111], in which recall of the critical set was hindered in the tested-last conditions (two right colum clusters in Figure 4B). However, recall was hindered across the board in condition [33 same] when the canonical order of presentation and test (*t*First-*p*First) was violated.

It is possible that our participants got confused about which set was being tested in condition [33 same], especially when the order of presentation and test did not match. To examine effects of presentation and test order without this issue of response confusion we excluded condition [33 same] and reran a 2 × 2 × 2 ANOVA. This analysis only compared conditions [33] and [3111] across the possible orders of presentation and test. We found decisive evidence for an effect of the test order (BF_incl_ = 2.48×10^11^, η_p_^2^ = 0.63), the interaction between test and presentation order (BF_incl_ = 89.02, η_p_^2^ = 0.20), and the sets condition (BF_incl_ = 30.88, η_p_^2^ = 0.20). We did not find evidence for a main effect of presentation order (BF_incl_ = 1.06, η_p_^2^ < 0.01). According to this model, the recall of the critical set was hindered when it was tested last, and it was slightly worse when it was also presented last. The effect of the sets condition was that, overall, performance was slightly better in condition [33] than in condition [3111], across all order conditions. This latest finding indicates a beneficial effect of having fewer sets when the number of items is held constant, hence replicating results in Experiment 1.

#### Number of items recalled from other sets

We compared performance in the other sets to further assess a potential effect of interference based on item similarity. Only items in the noncritical set are considered in this analysis. If item similarity is critical in constraining WM capacity, then we would expect that the recall of items in other sets to be the highest in [3111], intermediate in [33], and the lowest [33 same]. The rationale is that, in condition [3111], items in other sets are unique of their stimulus type in a trial (thus not prone to similarity-based interference), besides being presented with pauses as more distinct elements. In [33], each item representation suffers from interference from other two items of the same stimulus type. In [33 same], each item representation suffers from similarity-based interference from other five items of the same stimulus type.

A 3 × 2 × 2 ANOVA revealed that the best model accounting for recall in the other sets is the full model (BF = 6.71×10^41^), with decisive evidence for the inclusion of the sets condition (BF_incl_ = 1.55×10^23^, η_p_^2^ = 0.64), the test order (BF_incl_ = 1.42×10^10^, η_p_^2^ = 0.62), the presentation order (BF_incl_ = 1.73×10^3^, η_p_^2^ = 0.29) and their triple interaction (BF_incl_ = 3.05×10^5^, η_p_^2^ = 0.18). While condition [3111] (the one with the lowest level of similarity) provided no clear advantage for memory in the critical set when the order of presentation and test was preserved, memory in this condition was substantially better when recalling the other sets.

### Discussion

In Experiment 2, the number of items was equated across conditions and the input-output distances were varied to control for interference. We found no evidence that the number of interfering events per se hampered recall of the critical Set A, but rather a very strong deleterious effect of the test order, making performance in the critical set drop when it was tested last. Evidence for an effect of the order of presentation, according to which the first presented set was slightly benefited, was not found after eliminating the [33 same] condition that introduced ambiguity between the sets. Our results indicate that the locus of interference effects was at response output, an observation that is not novel in the field (Smith et al., 1970; Tulving & Arbuckle, 1966). Regarding the effects of set similarity, the comparable conditions in Experiment 2 (*p*First-*t*First) replicated Experiment 1, with no evidence for a deleterious effect. Moreover, we also replicated the finding that similarity matters for recall of items in the other sets, with lowest similarity yielding higher recall rates. In sum, the results of Experiment 2 concur with the findings of Experiment 1 and reinforce our interpretation of a general storage limit—rather than the number of interfering events—acting to constrain WM capacity.

## Experiment 3

Next, we explore another factor present in our Experiment 1 that could confound our results: the presence of pauses between sets, which could be critical for grouping. Alternatively, presenting items within sets in adjacent serial positions, hence in temporal contiguity, could be sufficient to yield similar results, including evidence for *H*_3_ (a limitation of both the number of items and the number of sets) and the efficiency trade-off between recall in Set 1 and in other sets).

The presence of pauses between sets in Experiment 1 could have induced participants to group items in a set, therefore making our results not necessarily reflect the way information is naturally represented in WM. To tackle this issue, we abolished the longer intervals between the sets in Experiment 3. The design essentially reproduces Experiment 1 but without the pauses separating the presentation of each set. If results in Experiment 1 were confounded with temporal grouping effects, then they should not be replicated in Experiment 3, in which the temporal grouping cue was omitted. Moreover, it is possible that pauses are essential for participants to chunk sets of items, so that the effect of the number of sets observed in Experiment 1 would be abolished in Experiment 3.

### Method

#### Participants

Sixty participants (*M*_age_= 26.8 years, *SD* = 2.9 years, 26 women, 22 men, 12 nonbinary or nondeclared) took part in Experiment 3 on Prolific, and none of them had previously taken part in Experiments 1 and 2. All participants were included in the analysis.

#### Stimuli, design, and procedure

Stimuli, design, and procedure were identical to Experiment 1 except that there were no pauses between each set. Items were sequentially presented for 250 ms with an ISI of 500 ms. The instruction screens with the trial structure were modified accordingly, with no spatial gaps representing the pauses between the sets; instead, a black dot between sets signaled the presence of items of different stimulus types in a trial (Fig. S3 in the supplementary materials)

#### Data analysis

The same scoring system and analyses as in Experiment 1 were applied to Experiment 3 data. We ran one-way ANOVAs with the factor condition to compare recall in Set 1 and in other sets. To test for differences between Experiments 1 and 3, we also ran mixed ANOVAs with the experiment as a between-subject factor and condition as a within factor.

### Results

#### Number of items recalled from Set 1

We found decisive evidence for a condition effect (BF_10_ = 6.01×10^62^, η_p_^2^ = 0.66), with conclusive evidence for an effect of the number of items. Set 1 recall was reliably better in conditions with fewer total items, with higher performance in [3] over all the other conditions; in [31] over [33], [33 same], [3111], and [3333]; and in [31], [33], [33 same], and [3111] over [3333] (all BFs > 90,000). There were no differences between conditions with the same number of items, so that [33] = [33 same] = [3111] (all BFs < 1.45). Finally, set similarity did not hinder recall of Set 1 (BF_10_ = 1.44), hence we found no evidence for a similarity effect. Again, these results concur to what was observed in Experiment 1. However, contrary to Experiment 1, we did not observe an advantage of having fewer sets in a trial when the total number of items was equated (BF_10_ = 0.16).

In a mixed ANOVA comparing Experiments 1 and 3, we found no evidence for a main effect of the experiment (BF_incl_ = 1.3). There was decisive evidence for the main effect of condition (BF_incl_ = ∞) and moderate evidence for an interaction between condition and experiment (BF_incl_ = 3.08), according to which recall of Set 1 in conditions [33] and [33 same] was slightly higher in Experiment 1. This is likely because of the longer pauses between sets in Experiment 1. In sum, the results of all paired comparisons observed in Experiment 1 were replicated in Experiment 3, except for the advantage of condition [33] over condition [3111]—thus not showing an effect of the number of sets.

#### Number of items recalled from other sets

**Fig. 5:**
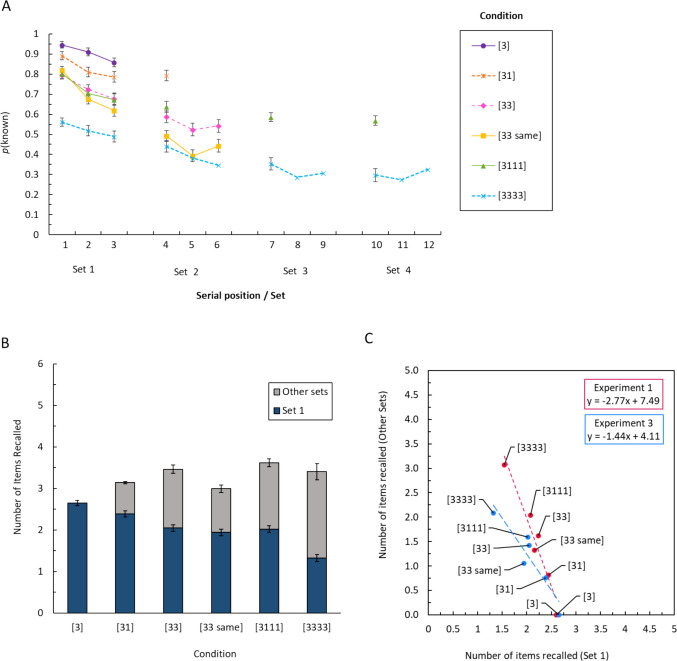
Performance in Experiment 3 and trade-off between recall in Set 1 and other sets, in Experiments 1 and 3. **A** Probability of knowing an item (*p*(known)) in each serial position of each experimental condition in Experiment 3. **B** Number of items recalled from Set 1 and from other sets, per condition of Experiment 3; the stacked columns represent the total number of items recalled across sets in a trial. Vertical bars represent standard errors. **C** Linear regression between the number of items recalled in Set 1 and in other sets in Experiments 1 and 3. Notice the more efficient trade-off between recall in Set 1 and other sets in Experiment 1, when sets were separated by pauses. (Color figure online)

**Fig. 6:**
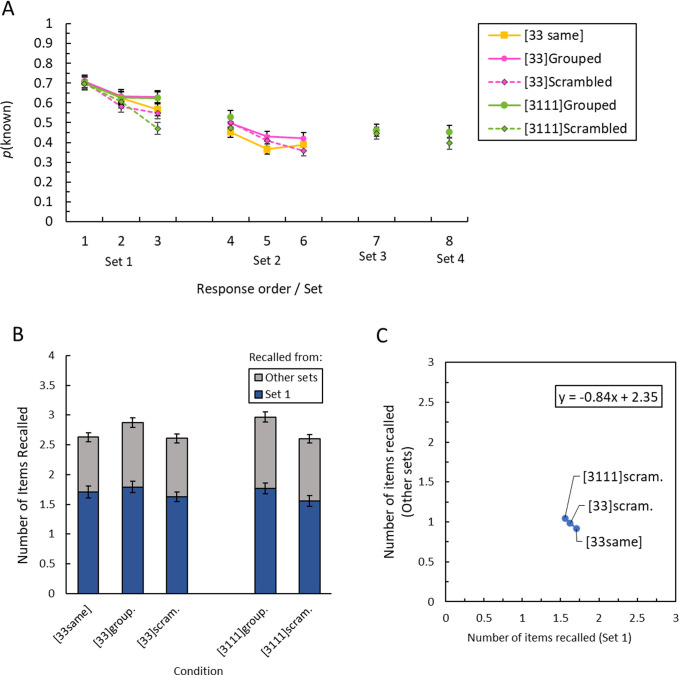
Performance in Experiment 4 and trade-off between recall in Set 1 and other sets, in scrambled conditions of Experiment 4. **A** Probability of knowing an item (*p*(known)) in each response, per condition in Experiment 4. The *x*-axis corresponds to the order of responses, so that 1 = first response given, 2 = second response given, so on and so forth. **B** Number of items recalled from Set 1 and from other sets, per condition of Experiment 4; the stacked columns represent the total number of items recalled in a trial. **C** Linear regression between the number of items recalled in Set 1 and in other sets in scrambled conditions of Experiment 4. (Color figure online)

We found decisive evidence for an effect of the experimental condition (BF_10_ = 1.31×10^20^) upon recall of the other sets, with differences in each paired comparison (all BFs greater than 6.45). However, this time the ANOVA comparing conditions across Experiments 1 and 3 revealed decisive evidence for a main effect of the experiment (BF_incl_ = 2.74×10^4^), with lower performance in Experiment 3. We also found compelling evidence for the interaction between experiment and condition (BF_incl_ = 1.63×10^4^), driven by an even larger advantage in condition [3333] of Experiment 1 compared to Experiment 3. In sum, the pattern observed for the recall of other sets was reproduced in Experiment 3 but with lower performance caused by the absence of pauses between sets; recall in condition [3333] was especially hindered by this factor in Experiment 3 (cf. Figs. 4A and 6A). Figure 5B depicts the mean number of items recalled from Set 1 and from other sets in Experiment 3. The full descriptive table of results in Experiment 3 can be found in the supplementary materials (Table S2), as well as a plot comparing Experiments 1 and 3 (Fig. S4).

#### Trade-off between recall of Set 1 and other sets

Repeating the analysis from Experiment 1, we looked into the relationship between the number of items recalled from Set 1 and from other sets in order to investigate the theoretical possibility of a trade-off between storage in the FoA and in aLTM. In Experiment 3, we also observed a negative linear relationship, but this time with a much less efficient trade-off: an increment of only about 0.44 item in the other sets per loss in Set 1. Figure 5C represents the linear regression between recall in Set 1 and in other sets in both experiments.

### Discussion

Experiment 3 was designed to test the hypothesis that temporal grouping modulated the effect of the number of sets in recall of Set 1 in that experiment. This hypothesis was confirmed. Although we replicated the strong effect of the number of items and the absence of a set similarity effect upon recall of Set 1, we did not replicate the effect of the number of sets. In Experiment 3, conditions with equal numbers of items yielded similar performances on Set 1 recall (all BFs < BF_10_ = 0.15). This finding in combination with results in Experiment 1 suggests that a qualification of *H*_3_ is needed. It is true only when there are pauses between sets, as was the case in Experiment 1. Nevertheless, we stress that the pauses do not modulate the effects of the number of items and set similarity, hence our conclusions for *H*_1_ and *H*_4_ remain the same: first, larger numbers of items hamper recall of Set 1; second, set similarity does not affect recall of Set 1. In the case of other sets, all paired comparisons replicated the results in Experiment 1, but with somewhat lower accuracy rates, especially in conditions with larger sets.

When looking at the relationship between recall in Set 1 and other sets, the linear trend suggests a much less efficient trade-off in Experiment 3 than in Experiment 1 (Fig. 5C). Together, the two experiments show that the pauses were crucial for the effect of the number of sets and the efficient trade-off between Set 1 recall and other sets recall. This suggests that time, or at least a clear boundary between sets, is key for the efficient trade-off to occur. Two possible phenomena can account for the advantage enabled by pauses: temporal grouping and chunking. In the former case, pauses increase the temporal distinctiveness of other sets, an additional retrieval cue that can be helpful at recall (Brown et al., 2007). In the latter case, pauses might have provided participants with more opportunities to chunk items within a set, especially those formed by triads; if chunking is successful, retrieving the first item of a set should enable retrieval of the whole set (Thalmann et al., 2019). In the next section, we explore another temporal variable that may be a boundary condition for the efficient trade-off: the organization of stimuli into regular series of the same item type in adjacent serial positions, or irregular series of mixed items in non-adjacent serial positions.

### Experiment 4

Besides the pauses, another characteristic of Experiment 1 might contribute to the grouping or chunking of sets: the presentation of items of the same set in adjacent serial positions. This organization makes the trial structure regular and predictable, with items in Set 1 always being reliably presented in serial positions 1, 2, and 3. We explored the role of this regularity in Experiment 4.

We manipulated the organization of the items within sets, so that they could be either presented in adjacent serial positions (grouped condition) or non-adjacent serial positions (scrambled condition). If the chunking of Set 1 also depends on its items being presented in adjacent positions, then we would expect to observe a strong advantage in grouped conditions. Alternatively, if chunking can occur based uniquely on the featural similarity of items in a set, then no differences between grouped and scrambled conditions should be observed.

#### Method

##### Participants

Sixty participants (*M*_age_ = 25.06 years, *SD* = 2.93, years, 35 women, 22 men, four nonbinary or nondeclared) took part in Experiment 4 on Prolific, and none of them had taken part in the previous experiments. All participants were included in the analysis.

##### Stimuli, design, and procedure

Stimuli were identical to the ones in previous experiments. We included only conditions of the same number of items (i.e., conditions [3111], [33], and [33 same]) and we manipulated the organization of items in Set 1, so that they could be either presented in a grouped or scrambled way. In grouped conditions, items in Set 1 were always presented in adjacent serial positions, as in previous experiments (e.g., in condition [3111], the order of presentation was A_1_A_2_A_3_B_1_C_1_D_1_). In scrambled conditions, items in Set 1 were presented in non-adjacent serial positions (e.g., in condition [3111], one possible order of presentation was A_1_B_1_A_2_C_1_D_1_A_3_). Therefore, set membership in scrambled conditions was uniquely defined by stimulus type (color, character, location, and shape), whereas it was defined both by stimulus type and item vicinity in grouped conditions.

There were five conditions in the experiment: [33]_*grouped*_, [33]_*scrambled*_, [3111]_*grouped*_, [3111]_*scrambled*_, and [33 same], for which the organization into the grouped or scrambled condition does not apply. In condition [33 same], participants were presented with a sequence of six items of the same stimulus type but tested separately for Set 1 (comprising the first three items in the sequence) and Set 2 (comprising the three latter items in the sequence). In scrambled conditions, the first item in the sequence always belonged to Set 1, and sets were always tested in the same order of presentation. For example, in condition [3111]_*scrambled*_, for a trial like A_1_B_1_A_2_C_1_D_1_A_3_, the participant was tested Set 1 (select items A_1_, A_2_, A_3_ from the pool of eight response choices), then in Set 2 (select item B_1_ among the eight choices), Set 3 (then select item C_1_ among the eight choices), and in Set 4 (and select item D_1_ among the eight choices). Table S3 in the supplementary materials describes all possible orders of stimuli presentation in each condition of Experiment 4. Figure S5 in the supplementary materials depicts an example of trial in scrambled conditions.

There were no pauses between sets, however this time participants were not warned about the trial structure before each trial. This was meant to avoid the predictability yielded by the structure layouts. Conditions were manipulated between trials. There were 20 trials per condition, totaling 100 trials; the experiment was preceded by five example trials in which no response was required and by five practice trials. Response feedback was given only in practice trials.

##### Data analysis

The data were corrected for guessing and the key analysis regards performance in Set 1, as previously. We ran a one-way repeated-measure Bayesian ANOVA to compare conditions. Moreover, we ran a 2 × 2 ANOVA to further explore the role of the number of sets ([33] or [3111]) and the sets organization (grouped or scrambled).

#### Results

##### Number of items recalled from Set 1

We found decisive evidence for the effect of condition (BF_10_ = 7.75×10^3,^ η_p_^2^ = 0.13), with post hoc comparisons showing superior performance in grouped than in scrambled conditions (all BFs > 13). There was no clear disadvantage in condition [33 same] in comparison to other conditions, as would be expected in a purely similarity account of WM capacity. On the contrary, we even found robust evidence (BF_10_ = 10.331) of an *advantage* in condition [33 same] compared to condition [3111]_*scrambled*_. Note that all items belong to the same stimulus type in [33 same] and that condition [3111]_*scrambled*_ has half as much similarity in the memoranda but without a clear grouped structure of sets. This suggests that the absence of an easily groupable configuration of the material can be more deleterious to recall than a high level of item similarity in itself.

The advantage of a grouped structure was confirmed in 2 × 2 ANOVA comparing the sets ([33] and [3111]) and organization (scrambled and grouped) conditions. Note that condition [33 same] was not included in this analysis because the factor organization does not apply to it. We found decisive evidence for the inclusion of the organization in the model (BF_incl_ = 6.67×10^3^, η_p_^2^ = 0.32), but not for the inclusion of the sets condition (BF_incl_ = 0.42, η_p_^2^ = 0.05). There was no evidence for an interaction either (BF_incl_ = 0.25, η_p_^2^ < 0.01). Contrary to what was observed in Experiment 1, conditions with fewer sets did not yield any advantage for Set 1 recall. Figure 6A shows the number of items recalled from Set 1 in each condition of Experiment 4, and Table S4 in the supplementary materials contains the descriptives.

##### Number of items recalled from other sets

Performance in grouped conditions was also superior to scrambled conditions for recall from other sets (BF_incl_ = 12.14, η_p_^2^ = 0.15). However, there was anecdotal to moderate evidence for an effect of the sets condition (BF_incl_ = 2.80, η_p_^2^ = 0.11), with [3111] yielding higher performance than [33] (BF_10_ = 3.93). This replicates the findings in Experiments 1 and 3 and reinforces our interpretation that the level of similarity in the memoranda matters for the later encoded items, due to proactive interference. There was no interaction between the factors sets and organization (BF_incl_ = 0.34, η_p_^2^ = 0.01).

##### Trade-off between recall of Set 1 and other sets

Contrary to what was observed in Experiments 1 and 3, there was no efficient trade-off between recall from Set 1 and from other sets in Experiment 4. Specifically in conditions without a clear grouped structure (i.e., scrambled conditions and condition [33 same]), the linear regression between the number of items recalled in Set 1 and in other sets showed a multiplicative term of only 0.84, meaning that for every forgotten item in Set 1, only about one item was recalled in the other sets. In the absence of a grouped structure and pauses between the sets, the rapid chunking of sets did not seem to occur in WM.

## Discussion

In Experiment 4, there was no evidence of chunking of sets in the scrambled conditions, with no efficient trade-off between the number of items recalled from Set 1 and from other sets. Participants not only seem to use pauses (Experiment 1), but also the grouped presentation in adjacent serial positions (Experiment 3 and grouped conditions of Experiment 4) to chunk items within sets. Additionally, Experiment 4 shows that simply being tested on items belonging to a categorical structure (here the four possible stimulus types) is not enough for beneficial grouping to occur. Mere set membership, as defined by items pertaining to the same stimuli type, did not seem to be sufficient for participants to chunk items of the same set. Rather, chunking of sets seems to require an easily groupable structure at encoding and enough time between sets.

Still on the role of feature similarity, we emphasize that Set 1 recall in condition [33 same] was not worse than in any other condition, and even surpassed recall in condition [3111]_*scrambled*_. We believe that, even without the pauses between sets, condition [33 same] still allowed participants to somehow organize the six items into two groups of three items each, boosting recall of Set 1. Condition [3111]_*scrambled*_, on the other hand, seems to have prevented any type of grouping of items in Set 1, and therefore yielded worse performance. This reinforces our interpretation that the presentation of items in a grouped structure is a predominant enabling factor for chunking, rather than feature similarity per se. Moreover, this result suggests that, at least for the sequential presentation of categorical visual information, feature similarity does not seem to be a decisive constraining factor of WM capacity.

## General discussion

This study addressed the question of which factors limit capacity when multiple sets of materials are to be held at once in WM: the number of items, the number of distinct sets, and/or similarity between sets. These capacity constraints are analyzed primarily in terms of recall of the first group (Set 1), which is the same across conditions but differs between conditions in competition from the various loads imposed by the concurrent maintenance of other sets during recall. Below, we summarize our findings and present a theoretical interpretation based on a dual-level storage system. Specifically, we propose that sets needed later in the trial are offloaded from the FoA to aLTM with some rapid learning of sets, and remain therein until they become relevant for response, at which time they are returned to the FoA and unpacked for recall. The sets in aLTM must be indexed by pointers that compete for storage in the FoA with pointers to currently relevant individuated items in the set being recalled. These pointers (cf. Awh & Vogel, 2025; Cowan, 2011) are conceived as abstract, contentless entities that index information held in aLTM so that it can be retrieved later in the trial.

### Summary of findings

Across experiments, for recall of Set 1, we observed both a general item limit and a limit based on the number of presented sets in WM when grouping in the temporal domain was possible (expressed in *H*_*3*_). Moreover, we found no deleterious effect of inter-set similarity for recall of Set 1. When there was a clear grouped structure (cf. Frankish, 1985; Hitch, 1996; Parmentier et al., 2006; Ryan, 1969a, 1969b)—namely, in Experiment 1, recall of Set 1 was better when the remaining items were organized into fewer sets (in particular, in condition [33] compared with condition [3111]). The clear, grouped presentation of Experiment 1 also enabled an efficient trade-off between recall of Set 1 and other sets, by which the loss of an item from Set 1 was compensated by the gain of more than one item from other sets. However, the benefit of fewer sets and the efficiency of trade-off were greatly reduced when clear grouping cues were removed, in Experiments 3 and 4 (e.g., see Fig. 5C). Variation of the order of presentation and test in Experiment 2 showed that our pattern of results was not a mere interference effect dependent on the input-output distance (see Bartsch & Oberauer, 2023).

One remarkable aspect of our results is that there can be both a benefit and a drawback of having multiple sets. This occurred in the comparison of the [33] and the [3111] conditions in Experiment 1, in which full grouping cues were present. The inclusion of four separate sets in the [3111] condition reduced recall of the first set, presumably because the maintenance of pointers for those sets interfered with Set 1 recall. Yet the dissimilarity between those sets allowed recall of the other sets in the [3111] condition that was better than recall of the second set in the [33] condition (Fig. 3).

Last, there was evidence against a deleterious effect of similarity upon recall of Set 1, in that performance was no worse when all sets had the same stimulus type (condition [33 same]) than when sets had differing stimulus types (condition [33]). These results indicate that capacity theories that are based on interference between similar items (e.g., Oberauer & Lin, 2017) may have validity in a limited domain. However, sameness of the two sets in [33 same] did interfere with recall of the second set in Experiments 1 and 3. Thus, output interference (Cowan et al., 2002) may be combined with set similarity to influence recall.

The key role of output interference in constraining recall is highlighted by another two findings in our study: first, the drop in performance in the tested-last conditions of Experiment 2; second, the absence of recency effects throughout all experiments, as illustrated by the serial position curves (Figs. 3A, 5A, and 6A). The recency effect manifests through a recall advantage of the final portion of a list, giving rise to a classic U-shaped serial curve—which was not observed here. Our interpretation is that when participants are forced to start retrieval by Set 1, output interference suppresses recency by hurting the final items in the sequence (i.e., in the later sets). This interpretation concurs with results by Cowan et al. (2002) for the recall of lists of nine visually presented digits. When participants recalled lists from the beginning, as in the ordinary procedure for serial recall, a very small recency effect was observed (Cowan et al., 2002, Fig. 2, upper panel). However, the recency effect was magnified when participants were prompted to start their responses from the end segment (Cowan et al., 2002, Fig. 3, leftmost panel). The opposite pattern emerged when the end segment was reported last (Cowan et al., 2002, Fig. 3, rightmost panel): here, no advantage is observed for the final items in the list.

In sum, our results support a chunk-based limitation framework when grouping conditions are warranted, with recall being also dependent on chunk size. When sets are not fully unitized into single chunks, the number of constituent items within those sets determines the effective load. Under these conditions, apparent item-based limitations emerge. Thus, overall, Hypothesis 2 is supported but with the extent of chunking greatly dependent on encoding conditions. The better the chunking (best in Experiment 1), the more efficient the trade-off, whereby more items are gained for each item lost in Set 1. Finally, we find no clear role for set similarity when output interference at retrieval is minimized, favoring a domain-general view of the capacity limit (Cowan et al., 2024; Wennberg & Serences, 2024).

### Theoretical interpretation

Our study shows the great power of grouping and, we believe, strongly suggests new chunk formation in allowing organized sets incorporating up to 12 items to be recalled with an efficiency beyond what would be expected in the case of homogeneous, ungrouped lists of so many items. The theoretical interpretation has to be one that permits both the severe limit of WM in many situations, such as recall of up to a few items from a homogeneous array (e.g., Luck & Vogel, 1997) or other procedures in which rehearsal and grouping are minimized (Cowan, 2001), but also astounding performance, such as an individual who was able to use known chunks and an hierarchical organization of chunks to raise his digit span to about 80 items after a year of practice (Ericsson et al., 1980) or the human ability to search for a large number of familiar objects concurrently (Wolfe, 2012). Our embedded-processes approach can handle this wide range of outcomes in that the FoA is limited to just a few pointers, whereas aLTM is unlimited except for material-specific interference between concurrently represented items (e.g., Oberauer & Awh, 2022) and temporal decay (e.g., Ricker et al., 2020), at least if items were not well-encoded (cf. Oberauer & Lewandowsky, 2008, for absence of decay when known objects can be encoded individually). The outcome of an experiment in terms of capacity depends on whether the situation allows a few pointers in the FoA to retrieve only a small amount of information from aLTM, given limits in what can be learned, or allows more to be learned with efficient chunking and organization and then retrieved.

**Fig. 7:**
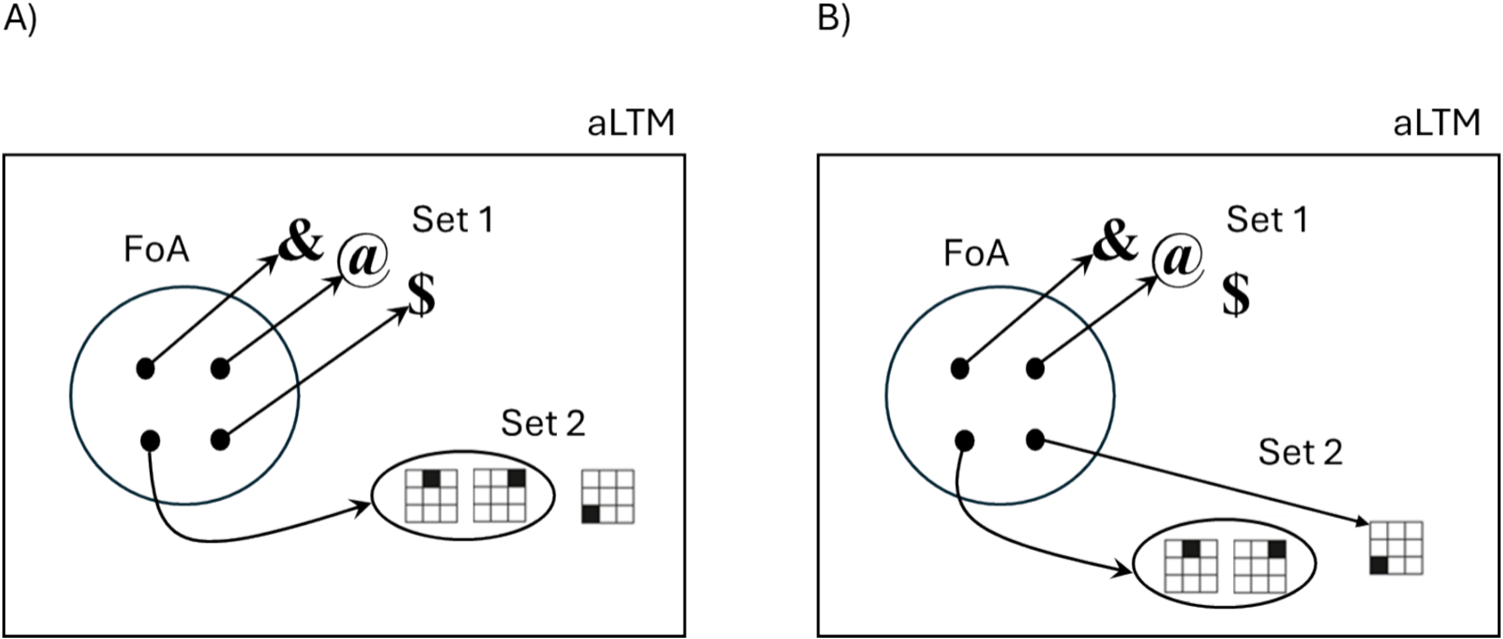
Proposal of storage competition between item representations and pointers in the focus of attention. Two possible pointer structures while recalling the first-presented set, in this example characters. In the example in (**A**), characters have been unpacked for recall, requiring one pointer each during their recall even if they required only one pointer as perfectly chunked set during retention. Because all three characters are indexed, recall of Set 1 will be perfect. In this example, the second set, locations, has been encoded as two chunks, thus requiring two pointers. However, because only the two-item chunk is being indexed, one location will not be successfully recalled. In the example in **B**, only two characters have been unpacked. These two characters will be recalled whereas the third activated character, the ampersand, has no pointer and is not successfully recalled. In this example, the second set, locations, has been encoded as two chunks, both of which are indexed by a pointer, thus taxing the limit in available pointers in the FoA. In principle, the presentation of multiple sets can tax the recall of either set. The assumption of a capacity of 4 pointers is only approximate and presumably varies between individuals

Holding a pointer in the FoA should take no more than one slot. For example, assume a fixed capacity of four slots in the FoA. While Set 1 is retrieved, three slots in the FoA could store pointers to each of the three individuated items of Set 1, represented in aLTM, and leaving one free slot to hold a pointer to the additional sets in aLTM; this pointer could index either a single-item chunk or a multi-item chunk. In this case, recall of Set 1 is perfect to the detriment of recall of the other sets. In a second scenario, only two slots are used to hold pointers to items in Set 1 in the FoA, the rest of the capacity being allocated to holding two pointers to items in other sets; here, recall of other sets is superior to the detriment of recall of Set 1. We illustrate these two possibilities in Fig. 7.

In this conception, each newly-learned chunk may require only one pointer in the FoA while the chunk itself is retained in aLTM. However, when the chunk is to be retrieved, it presumably has to be unpacked into the FoA (Huang & Awh, 2018; Rhodes & Cowan, 2018; Thyer et al., 2022; Wickens et al., 1981), requiring more slots in the capacity-limited region during its unpacking and recall; unpacked chunks in the FoA would compete more heavily with pointers to other chunks during recall than during its maintenance.^2^The two-level storage via a capacity-limited FoA and storage of pointers to information in aLTM is based on conditions in which sets are clearly marked by grouping cues, as in Experiment 1. Under those conditions, it can account for many of our results. First, it accounts for the capacity limitation imposed on Set 1 based primarily on the number of items but secondarily on the number of sets into which the items are organized. Second, it accounts for the efficient trade-off in which more items are gained from other sets than are lost from Set 1, because pointers can index single or multi-item chunks for the same capacity cost. This point is nicely illustrated by studies showing equivalences between chunked ensembles and individual items of various types in load-specific CDA signals (Balaban et al., 2019; Rajsic et al., 2019; Thyer et al., 2022; Woodman & Vogel, 2008). Finally, it accounts for the no deleterious effect of set similarity upon recall of Set 1, because of the abstract nature of pointers held in the FoA for other sets during recall of Set 1.

The theory also seems suitable to explain results of Uittenhove et al. (2019), who presented lists of visual and auditory letters for recall and varied the number of items presented in each modality within a list. In their Fig. 7, they showed a somewhat comparable trade-off phenomenon occurs with verbal material. For lists with three visual letters, recall of those letters decreased from about .90 when there were only two auditory letters in the same list, to about .78 when there were four auditory letters, to about .55 when there were six auditory letters. By transforming these recall rates to the number of letters recalled (e.g., set size 3 × accuracy .90 = 2.7 letters recalled), participants recalled about 2.7, 2.3, and 1.7 visual letters, respectively. Meanwhile, the acoustic letters recalled from those same lists increased from about 1.9 to 3.6 to 4.0, respectively. In sum, a sacrifice of about 1.0 visual letter was rewarded by the addition of about 2.1 auditory letters in their study. This example lends further encouragement to the notion that, with the right coding, loss of information from WM for one set can be compensated by a greater number of items gained from other sets.

The finding that the benefits of grouping occur only with clear marking of sets extends also to recent work on the Hebb repetition effect by Musfeld et al. (2024). In their study, the chunking of the repeated sequence occurred regardless of its placement within a list (e.g., at the start, the middle, the end, or at varying consecutive serial positions), as long as the repeated structure was easy to detect, and items were presented in vicinity (Experiments 1–4). By means of presenting the repeated items in a salient color but sometimes in nonadjacent serial positions, the authors disentangled the saliency of the repeated structure from item vicinity (Experiments 5 and 6). Hebb learning was drastically reduced when the repeated items were interspaced with filler items, even though the repeated structure was easily detectable—reinforcing our conclusions in our Experiment 4. A caveat must be made, though: their task (a classic recall paradigm with Hebb repetition) is not suitable for observing trade-offs between storage in the FoA and aLTM, and thus it does not have a bearing on our proposal that chunks in aLTM are indexed by pointers in the FoA.

In addition to an easy-to-detect grouped structure, pauses between groups bolster the efficiency of the trade-off between recall in Set 1 and other sets, as shown by the comparison between the linear regressions in Experiments 1versus 3 (Fig. 5C). In Experiment 1, when there is temporal contiguity between items in the same set and pauses between sets are included, the trade-off between Set 1 and other sets recall was 1:2.77. In Experiment 3, when temporal contiguity was maintained but pauses were removed, the trade-off was 1:1.44—still efficient, but nevertheless worse than in Experiment 1. In sum, an efficient trade-off can only take place if items are presented in a grouped structure allowing for rapid chunking to occur, and pauses appear to maximize this efficiency by allowing extra time for those newly-learned chunks to consolidate before being transferred to aLTM.

#### The mechanics of pointer assignment in WM

Here we propose that storing multiple sets of items in WM can only be possible by assuming that, in any particular time point during maintenance, the FoA contains pointers to offloaded information that is kept “on hold” in aLTM. Offloaded information in aLTM can be represented either as one- or multi-item chunks that were rapidly learned at encoding. In this dual WM architecture, the FoA contains pointers to individuated items in the set that is currently relevant, and also pointers to chunks not yet relevant for retrieval (Cowan, 2019; Huang & Awh, 2018; Rhodes & Cowan, 2018).

**Fig. 8:**
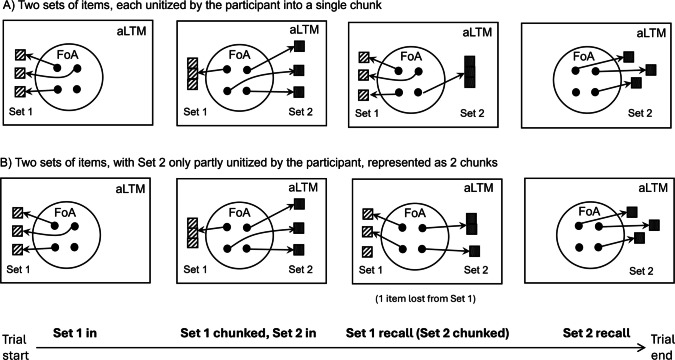
Proposal of how pointer indexing takes place during the trial. The figure depicts two examples in condition [33]. FoA = Focus of attention; aLTM = activated long-term memory. The dots in the FoA represent slots, arrows represent pointers, and the different textures represent item types in different sets. Offloaded sets in aLTM can be indexed by one or more pointers, depending on how many items are unitized into the new chunks. In Panel **A** both sets were perfectly unitized into a new chunk. In Panel **B**, Set 1 is perfectly unitized, but Set 2 is only partly unitized and thus indexed by two distinct pointers. This dual indexing of Set 2 comes at the cost of recalling of one item from Set 1. Whenever a set is held in awareness, item-specific information must be unpacked from the chunk, and each constituent item needs to be individually indexed by a single pointer

In our framework, the processes taking place during a trial could proceed as follows. Each set would be encoded into the FoA in turn and, as the trial progresses, the sets are rapidly chunked (albeit often imperfectly) and offloaded to aLTM and put “on hold” by the means of pointers, until they become relevant for recall. Every time a new set is encoded, the previous sets are offloaded to aLTM and indexed by pointers. At response, the first-tested set is unpacked and each of its items to be recalled then must be indexed by a separate pointer in the FoA, whilst pointers to the remaining sets are held until responses are given. Then the first set is discarded from WM as the second set is unpacked into the FoA and recalled, and so forth until all sets are tested in a trial. Thus, a key assumption is that item information in the later presented sets—not yet relevant for response—are not represented in the FoA during the test phase of previous sets except for a single pointer per chunk in aLTM. Take a trial in condition [33], for example. At the encoding of Set 1, three items are represented in the FoA (each with a pointer). Before the presentation of Set 2, the participant attempts to rapidly chunk Set 1 and offloads it to aLTM; if chunking is at least partly successful, they will hold fewer than 3 pointers for it while Set 2 is encoded into the FoA with further rapid chunking. During the test phase, Set 1 is unpacked and its three items are indexed by a pointer each in the FoA. At this time, Set 2 has been offloaded to aLTM and indexed by hopefully fewer than three pointers, which compete for storage in the FoA with pointers to single items in Set 1 until that set is recalled. Finally, Set 2 is unpacked and recalled. Figure 8 illustrates the proposal of storage competition in the FoA between pointers to items and pointers to chunks in aLTM.

#### The role of pointers in WM

We have noted in our discussion that pointers can be allocated to either singletons or multi-item chunks held in aLTM, depending on the presentation type and the degree of success in associating items within a group. From this point of view, a chunk is a chunk no matter its composition or extent, a view that was verified with findings that capacity has the same limit no matter whether the contents are single- or multiple-item chunks (Chen & Cowan, 2005, 2009; Cowan et al., 2004, 2012). This conception allows us to conceive of the FoA as holding contentless pointers that index the representation of chunks in disparate locations in aLTM (cf. Cowan et al., 2011). That conception eliminates the need to posit separate representations for items in and out of attention or to have two different levels of concept, chunks on one hand and pointers on another, directly compete for capacity. The pointer notion is consistent with an earlier description in which the FoA is viewed as a spotlight on attended representations (e.g., Cowan et al., 2005), except that it also facilitates a description of the capacity limit as a limit in the number of pointers held concurrently (equivalent to the number of chunks that can be spotlighted). Neurally, pointers would reflect the functional connectivity of the core of the FoA, which we believe to be the intraparietal sulcus, with representations in the posterior cortex, which we believe to be collectively aLTM based on evidence discussed earlier (for reviews, see Cowan et al., 2024, 2025).

If all pointers are comparable in form, the pointer concept also can help us to understand domain-specific and domain-general aspects of interference between items in WM. Several studies suggest that the encoding of items is susceptible to material-specific interference, with colors interfering most with other colors, orientations with other orientations, letters with other letters, and so on; but that WM maintenance following encoding is domain-general. In particular, there is material-specific encoding, but all types of memoranda interfere with each other equally following a retro-cue indicating which items to retain in WM (Cowan & Morey, 2007; Wennberg & Serences, 2024). These results can be explained if there is material-specific interference between representations of chunks in aLTM but, in the FoA, only domain-general interference (i.e., a capacity limit for pointers). Reinforcing that account, Oberauer and Awh (2022) found that subcapacity sets of up to four items did not show proactive interference from similar items presented in previous trials, presumably because pointers in the FoA are not susceptible to this kind of interference, whereas larger sets did show this interference, presumably because retrieval from aLTM (or what they referred to as episodic LTM) was necessary for those set sizes and is susceptible to material-specific proactive interference.

In an open-ended recall procedure, one role of the pointer would be to retain the serial order of sets. We, however, provide response options that indicate which set is being tested. In our procedure, the pointer still must be used to retrieve the set (e.g., of characters) presented on the current trial as opposed to similar sets presented on previous trials, allowing this set to be unpacked, temporarily taking up more pointers, one for each item from the set that is being recalled. Thus, the use of pointers is a process that should minimize interference from other multi-item sets during the recall of any given set in our procedure.

#### Are there competing, potentially simpler accounts?

It might be suggested that an attentional indexing system that relies on pointers to aLTM may not be needed to explain the item- and set-based limitations and efficient trade-off between Set 1 versus later sets observed when grouping cues were strong (Experiment 1). One alternative is that temporal grouping alone could explain the results (Liu & Caplan, 2020; Ryan, 1969a). Although temporal distinctiveness could explain the recall advantages observed in Experiment 1 compared with other experiments, it cannot accommodate the orderly trade-off of 1:2.77 items. This trade-off suggests that a nearly perfect triad (2.77 items) was recalled in exchange for one representation in Set 1, which we interpret as three items being chunked and subsumed by one pointer.

An account based on chunking alone would assume that items within a set (but not items from different sets) are chunked and that chunking operates via lossy data compression (Nassar et al., 2018; Norris & Kalm, 2021; Norris et al., 2020). However, in condition [3111] in Experiment 1, items in Set 1 compete with three singletons from other sets. Because three singletons occupy more space than a compressed chunk of three items, recall of Set 1 suffers in condition [3111] compared with condition [33], the opposite of what would be expected based on just the number of chunks to be remembered.

## Conclusions and future directions

We have shown the extent to which grouping improves memory for multiple, distinct sets of information and have suggested the role of newly learned chunks. In accounting for the findings, recent research has established the need for pointers indexing objects and mapping spatial locations in visual WM (e.g., Awh & Vogel, 2025; Balaban et al., 2019; Ngiam et al., 2024; Thyer et al., 2022). There also is a possibility of stand-in information that has content but refers to a larger chunk (e.g., a word to stand for a multi-item conglomerate; a title to refer to a song). Moreover, in keeping with Awh and Vogel (2025), a capacity-limited region of WM might hold only pointers, so that singletons still have pointers. A chunk that is represented by a single pointer in WM maintenance, when unpacked for recall, might require a separate pointer for each item within the chunk that is about to be recalled.

One of our reviewers raised an interesting question: how does our framework extend to the storage of simultaneous arrays of objects, as opposed to the sequential groupings in our paradigm? We tentatively propose that the same encoding and storage constraints would apply in scenarios involving multiple spatially distinct object groupings. Given that individuals tend to inspect visual scenes in localized regions (e.g., Noton & Stark, 1971), and considering the well-documented limitations on the amount of visual information that can be encoded at once (e.g., Bays & Husain, 2008; Craston et al., 2009; Zhang & Luck, 2008, 2011), we argue that only one group of objects is likely to be encoded at a time. Offloading and pointer assignment would need to take place so that another group of objects is encoded, so on and so forth, until retrieval. From an experimental standpoint, it would be necessary to control for test order (e.g., by testing the groups by quadrants of the screen). This remains to be explored in future studies.

In sum, through a novel paradigm testing memory for multiple sets of items, we have observed that materials consisting of organized, temporally separated sets of items of different types (shapes, colors, locations, and characters) produce WM capacities well beyond what one would expect for up to 12 items if they were homogeneous (Experiment 1). Recall depends not only on the number of items; there is a cost to the initial set to be recalled if the subsequent items represent multiple sets. There is an efficiency in recall in that one item lost from the first set to be recalled is traded for multiple items in later sets as the overall memory load increases. This pattern is not merely based on interference from the input-output distance (Experiment 2). The efficiency of recall is greatly reduced if the sets are not separated by pauses (Experiment 3) and is lost entirely if the groups are scrambled (Experiment 4).

We have argued that two regions of WM contribute to memory capacity here: an aLTM that holds newly-learned chunks, and a limited, central attentional portion (the FoA) that holds pointers to items immediately relevant for response and also retains pointers to chunks in aLTM to be retrieved later in the trial. Although details of the theoretical account need to be ascertained in further research, the pattern of results with several different stimulus types comprises a novel data base for a kind of situation that is common in daily life but has been absent from prior research.

## Electronic supplementary material

Below is the link to the electronic supplementary material.

Supplementary file1 (DOCX 738 KB)

## Data Availability

The data and materials for all experiments are available on OSF (https://osf.io/2abxs/); this study was not preregistered.

## Appendix A

### Decomposition of Proportion Correct into Knowledge and Guessing Contributions

1 $$\mathrm{p}\left(\mathrm{correct}\right)=\text{ p}\left(\mathrm{known}\right)+\text{ p}\left(\text{not known}\right)*\mathrm{p}\left(\mathrm{guess}\right)$$

In which:

2 $$\mathrm{p}\left(\mathrm{known}\right)=\frac{{\mathrm{k}}_{\mathrm{Si}}}{{\mathrm{SS}}_{\mathrm{i}}}$$

3 $$\mathrm{p}\left(\text{not known}\right)= 1 -\frac{{\mathrm{k}}_{\mathrm{Si}}}{{\mathrm{SS}}_{\mathrm{i}}}$$

4 $$\mathrm{p}\left(\mathrm{guess}\right)=\frac{1}{8 - {\mathrm{k}}_{\mathrm{Si}}}$$

So that:

5 $$\mathrm{p}\left(\mathrm{correct}\right)= \left(\frac{{\mathrm{k}}_{\mathrm{Si}}}{{\mathrm{SS}}_{\mathrm{i}}}\right)+ \left[\left(1 -\frac{{\mathrm{k}}_{\mathrm{Si}}}{{\mathrm{SS}}_{\mathrm{i}}}\right)*\left(\frac{1}{{8-\mathrm{kS}}_{\mathrm{i}}}\right)\right]$$

We did not use a closed-form solution for *k*_Si_ with this equation, but created a lookup table for *k*_Si_ corresponding to *p*(correct), with *k*_Si_ estimated in increments of .01. We also calculated the number of items recalled from the other sets by using the same formula, but this time by adding the *p*(known) of items across all the sets that succeeded Set 1. For example, in condition [3333], the number of items recalled from the other sets includes the probability of knowing items across Sets 2, 3 and 4. These measures were calculated per participant and averaged across experimental conditions. Further details on the logic behind our guessing corrections with concrete examples can be found in the supplementary materials.

## Footnotes

1 Throughout this manuscript, the term “focus of attention” (FoA) is used in line with the embedded-processes model of WM by Cowan (2001; Cowan et al., 2024), in which it is conceived as containing up to about three or four representations simultaneously. Therefore, we consider the FoA to have a capacity-limited storage property. Note that our use of the term contrasts with the proposal of a single representation being prioritized in the FoA at any given time point (cf. Oberauer, 2002, 2021); in the latter proposal, the FoA is conceived as a selecting mechanism rather than a storage resource. If one prefers to adopt Oberauer’s WM framework, then one can substitute “capacity-limited region” in place of our use of the term “FoA” for a similar effect.

2 One of our reviewers asked about the limits of aLTM in the proposed system, more specifically about the theoretical possibility of an infinite hierarchical organization of pointers to chunked information in aLTM (i.e., pointers to chunks of chunks, indefinitely). We think that such an infinite hierarchical storage in aLTM can only be conceivable in situations allowing for extensive training, as in skilled memory theory (Chase & Ericsson, 1982). For example, in a famous case described by Ericsson et al. (1980), an undergraduate student with average span and intelligence reached a span of nearly 80 digits after undergoing more than 230 hours of lab testing, spread over more than 1 year. The impressive span was reached by employing a hierarchical retrieval structure of three levels of supergroups divided into subgroups of up to five digits, and by making associations with dates, ages, and running times. In a sense, the pointer proposal can be seen as akin to hierarchically organized retrieval cues in that item information in a chunk is not held in the direct region of access, but rather rely on a controlled retrieval process that brings items back to the FoA. However (and importantly), we do not expect an infinite process assignment requiring extensive training to happen in the short period of our task, although this issue is beyond the scope of our theory.

## Declarations

### Author note

This study was conducted at the University of Missouri with support from NIH Grant R01-HD021338 to Cowan. The work was carried out while the first author was affiliated with the University of Missouri. Thanks to Bret Glass for assistance with data handling and Dominic Guitard for programming advice. We would also like to thank Ed Awh and Brad Wyble for their many insightful comments and feedback provided during the peer-review process. Study materials and data are available on OSF: https://osf.io/2abxs/
