# Supplementary material for: Colors, characters, locations, and shapes: The capacity of working memory for multiple, dissimilar sets of items
Source: Mem Cognit. 2026 Jan 14;54(4):1263–91. doi: 10.3758/s13421-025-01809-7 (PMC13253884; doi:10.3758/s13421-025-01809-7)
Supplement: Supplementary file 1 — Supplementary file1 (DOCX 738 KB) [file 13421_2025_1809_MOESM1_ESM.docx]

**Supplementary Material for:**

**Colors, Characters, Locations, and Shapes: The Capacity of Working Memory for Multiple, Dissimilar Sets of Items**

[**Additional Material for the Appendix** 3](#_Toc191999349)

[**Additional Material for Experiment 2** 4](#_Toc191999350)

[**Figure S1** 5](#_Toc191999351)

[*Example of a Trial in All Order Conditions of Experiment 2, in Condition [33]* 5](#_Toc191999352)

[**Table S1** 7](#_Toc191999353)

[*Mean Number of Items Recalled from the Critical Set A, Other Sets, and Across Sets, per Condition of Experiment 2* 7](#_Toc191999354)

[**Figure S2** 8](#_Toc191999355)

[*Relationship Between the Number of Items Recalled from the Critical Set A and from Other Sets of Experiment 2, in Conditions Comparable to Experiment 1, and Mean Number of Items Recalled from Other Sets in All Conditions of Experiment 2* 8](#_Toc191999356)

[**Additional Material for Experiment 3** 9](#_Toc191999357)

[**Figure S3** 9](#_Toc191999358)

[*Example of a Trial in Condition [33] in Experiment 3* 9](#_Toc191999359)

[**Table S2** 10](#_Toc191999360)

[*Mean Number of Items Recalled from the Critical Set A and Other Sets, per Condition of Experiment 3* 10](#_Toc191999361)

[**Figure S4** 11](#_Toc191999362)

[*Mean Number of Items Recalled from Set 1 and Other Sets in Experiments 1 and 3* 11](#_Toc191999363)

[**Additional Material for Experiment 4** 12](#_Toc191999364)

[**Figure S5** 12](#_Toc191999365)

[*Example of Trial in a Scrambled Condition of Experiment 4, Using Condition [33] as an Example* 12](#_Toc191999366)

[**Table S3** 13](#_Toc191999367)

[*Description of Conditions in Experiment 4.* 13](#_Toc191999368)

[**Table S4** 14](#_Toc191999369)

[*Mean Number of Items Recalled from Set 1, Other Sets, and Across Sets, per Condition of Experiment 4* 14](#_Toc191999370)

[**Normal Probability Plots of the Residuals (Q-Q Plots) for Aggregated Data** 15](#_Toc191999371)

[***Experiment 1, Set 1*** 15](#_Toc191999372)

[***Experiment 1, Other Sets*** 16](#_Toc191999373)

[***Experiment 2, Critical Set A*** 16](#_Toc191999374)

[***Experiment 3, Set 1*** 17](#_Toc191999375)

[***Experiment 3, Other Sets*** 17](#_Toc191999376)

[***Experiment 4, Set 1*** 18](#_Toc191999377)

[***Experiment 4, Other Sets*** 18](#_Toc191999378)

# **Additional Material for the Appendix**

The point of correcting for guessing is to estimate *k*, the number of items in working memory from a set of items. Consider how our formula plays out in sets of 3 items. In trials in which the correct choice for Serial Position *n* has not been used up in a response to a previous serial position, the number of choices has been reduced relative to the previous position; but in trials in which the response to Serial Position *n* has already been used up, the probability of a correct response to Serial Position *n* is zero. It turns out that these situations balance out so that the guessing rate is overall the same across serial positions. For example, imagine that one item out of three is known (*k* = 1). Then there are 7 (out of an initial 8) choices left to be used for two serial positions with unknown choices. The chance for the first unknown serial position to be answered correctly would be 1/7. Now consider the second such serial position. On 1/7 of the trials, the response to the first unknown serial position would have used up the choice that was correct for the second one, so the answer for the second unknown serial position will be incorrect for sure. On the other 6/7 of the trials, the correct response for the second unknown serial position is available and the probability of a correct response for that location is 1/6. Overall, then, the probability of getting the second unknown serial position correct is (6/7) x (1/6) = 1/7, which is identical to the chance for the first unknown serial position. Similarly, if *k* = 0, the first serial will be correct 1/8 by chance, the second serial position will be correct 7/8 x 1/7 = 1/8, and the third serial position will be correct on a proportion of trials in which its correct choice was not used up: (6/8) x (1/6) = 1/8. Other remaining possibilities are simpler: If *k* = 2 there is only one unknown location to consider (chance of correct guessing 1/6), and if *k* = 3 there is no guessing. Our formula encompasses all these possibilities.

# **Additional Material for Experiment 2**

We observed very strong evidence for the effect of the number of items presented in a trial upon recall of Set 1 in our Experiment 1. Experiment 2 tested for an alternative account of those results: that the limiting factor of recall was the number of interfering events between the presentation and test of Set 1, which was fully confounded with the number of items in a trial in that experiment. To test this account, we manipulated the order of presentation and test of the critical Set 1 (henceforth called Set A, to avoid confusion) so that some conditions entailed long, medium, and short input-output distances, and thereby high, medium, and low interference upon Set A, respectively. The input-output distance corresponds to the number of items presented after Set A and the responses given before Set A is tested. For this experiment, only conditions with the same set sizes were selected, i.e., conditions [33], [33 same], and [3111]. Figure S1 represents all possible orders of presentation and test of the sets, using condition [33] as an example.

## **Figure S1**

## *Example of a Trial in All Order Conditions of Experiment 2, in Condition [33]*

*
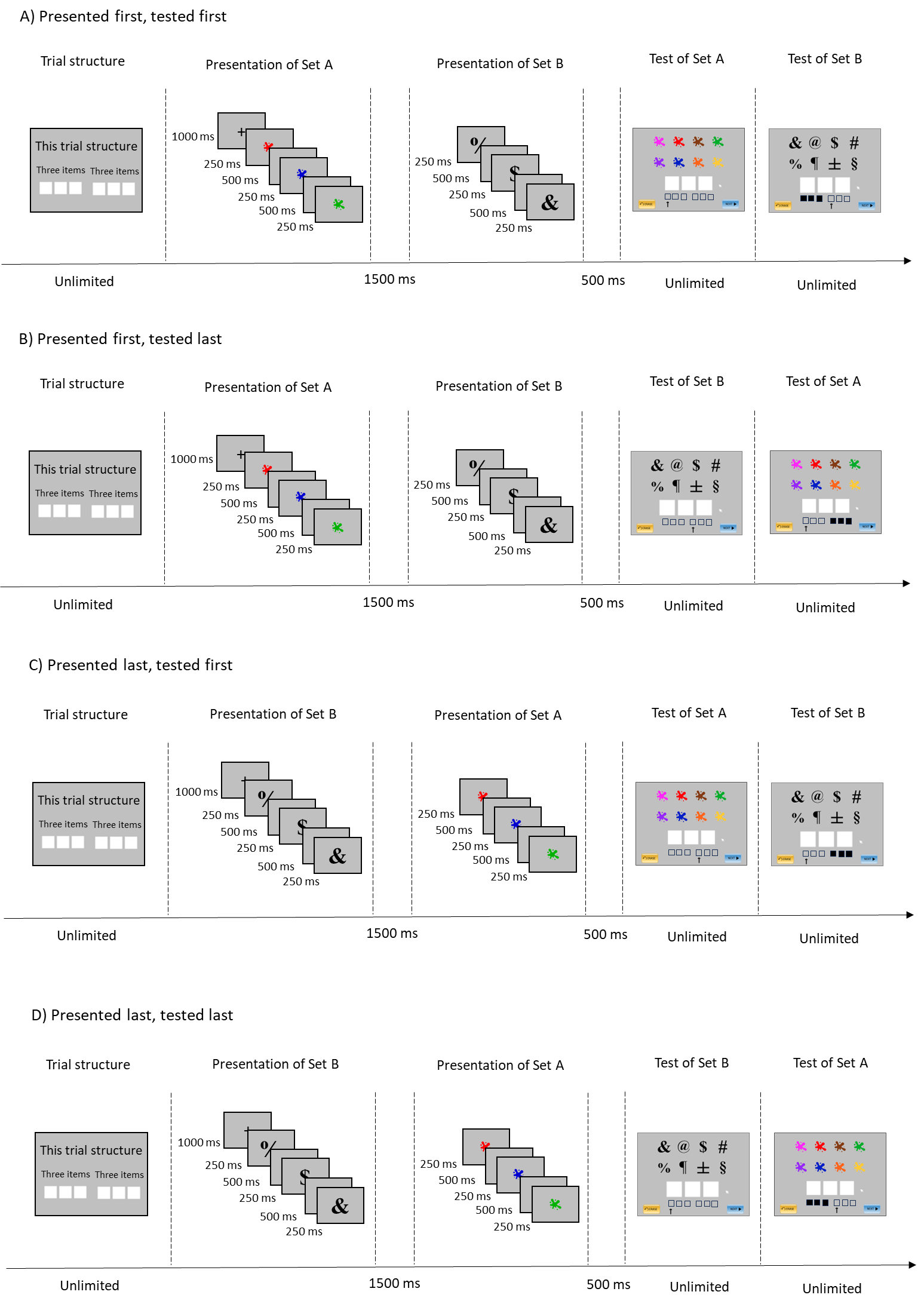
*

*Note.* The input-output distances are 3 in (A), 6 in (B), 0 in (C), and 3 in (D). The bottom part of the response screens informs which set is currently being tested and which responses have already been completed by the participant. The figure is not to scale.

Table S1 contains the complete descriptives of the number of items recalled from the critical Set A, the other sets, and across sets in each condition of Experiment 2. Note that the rows in bold indicate conditions that are comparable to the ones in Experiment 1, i.e., the items are presented and tested in forward order, with Set A being comparable to Set 1 in that experiment. Figure S1 depicts the relationship between the number of items recalled from the critical set A and other sets in the comparable conditions (panel A), and the mean number of items recalled from other sets in all order conditions of Experiment 1 (panel B).

## **Table S1**

## *Mean Number of Items Recalled from the Critical Set A, Other Sets, and Across Sets, per Condition of Experiment 2*

|  | *Order condition of the critical Set A* | | |
| --- | --- | --- | --- |
|  | Presented first – tested first | | |
| Sets condition | Set A | Other sets | Across sets |
| **[3111]** | **2.09 (0.08)**  **[1.92-2.25]** | **2.10 (0.09)**  **[1.93-2.27]** | **4.18 (0.16)**  **[3.87-4.49]** |
| **[33]** | **2.21 (0.07)**  **[2.06-2.37]** | **1.71 (0.10)**  **[1.52-1.91]** | **3.92 (0.15)**  **[3.614-4.23]** |
| **[33 same]** | **2.16 (0.08)**  **[2.00-2.33]** | **1.51 (0.09)**  **[1.33-1.70]** | **3.67 (0.16)**  **[3.36-3.98]** |
|  | Presented first – tested last | | |
| [3111] | 1.82 (0.09)  [1.63-2.00] | 2.35 (0.08)  [2.20-2.52] | 4.17 (0.16)  [3.86-4.48] |
| [33] | 1.97 (0.08)  [1.81-2.14] | 2.16 (0.10)  [1.97-2.36] | 4.14 (0.16)  [3.82-4.46] |
| [33 same] | 1.64 (0.09)  [1.46-1.83] | 1.57 (0.10)  [1.37-1.76] | 3.21 (0.18)  [2.85-3.56] |
|  | Presented last – tested first | | |
| [3111] | 2.16 (0.1)  [1.96-2.36] | 2.18(0.09)  [2.00-2.4] | 4.34 (0.17)  [3.99-4.68] |
| [33] | 2.21 (0.09)  [2.03-2.39] | 2.06 (0.08)  [1.91-2.22] | 4.27 (0.14)  [3.98-4.56] |
| [33 same] | 1.55 (0.10)  [1.35-1.76] | 1.60 (0.11)  [1.39-1.82] | 3.16 (0.20)  [2.75-3.56] |
|  | Presented last – tested last | | |
| [3111] | 1.56 (0.10)  [1.35-1.77] | 2.40 (0.07)  [2.25-2.56] | 3.96 (0.16)  [3.64-4.29] |
| [33] | 1.73 (0.07)  [1.56-1.91] | 2.32 (0.08)  [2.17-2.48] | 4.06 (0.15)  [3.76-4.35] |
| [33 same] | 1.42 (0.11)  [1.21-1.63] | 2.15 (0.06)  [1.97-2.32]] | 3.56 (0.18)  [3.02-3.92] |

*Note.* Values between parentheses are standard errors of the mean and values between brackets are the 95% credible intervals. The comparable conditions to Experiment 1 are in bold (cf. Table 4 in the main text for Experiment 1 results).

## **Figure S2**

## *Relationship Between the Number of Items Recalled from the Critical Set A and from Other Sets of Experiment 2, in Conditions Comparable to Experiment 1, and Mean Number of Items Recalled from Other Sets in All Conditions of Experiment 2*

*
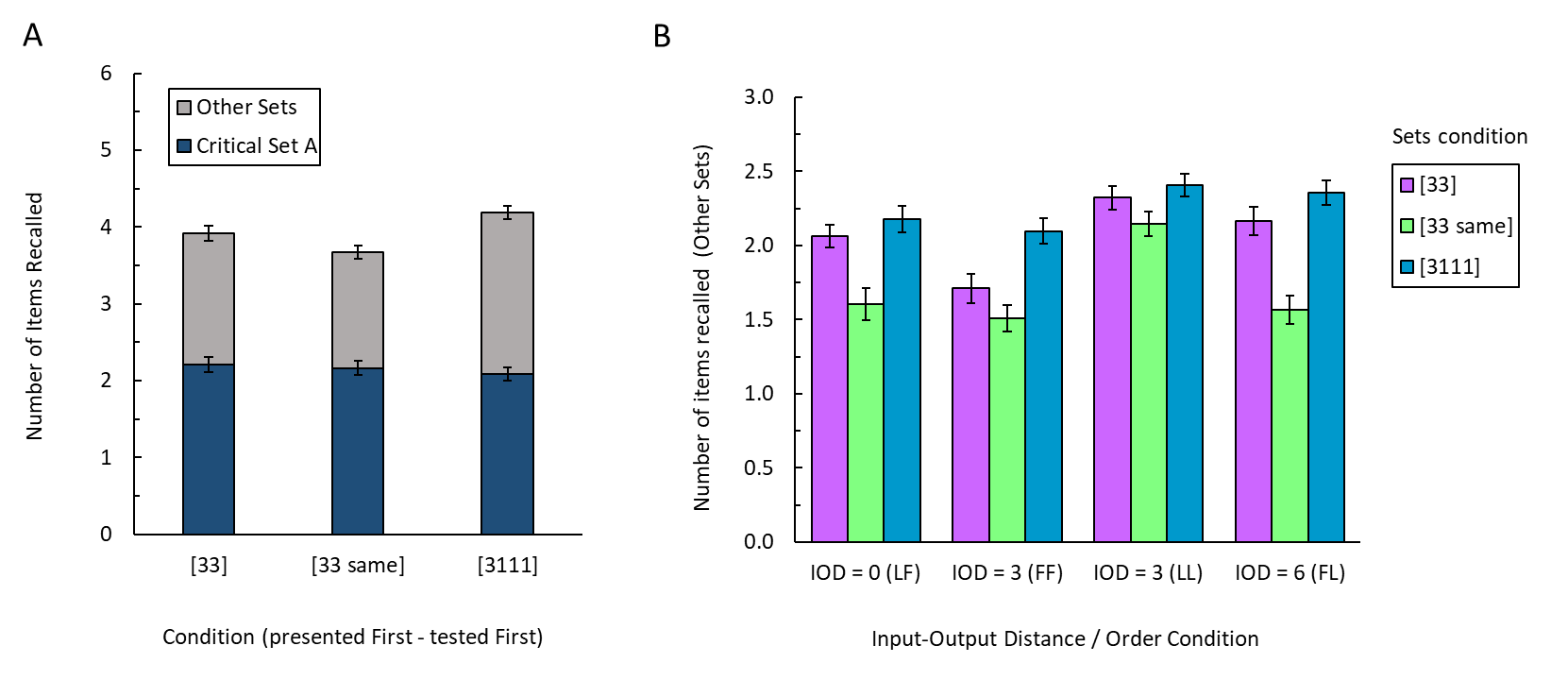
*

*Note.* Panel (A) depicts only order conditions that are comparable to the ones in Experiment 1, i.e., presented First – tested First. The stacked columns represent the mean total number of items recalled across sets in a trial. Panel (B) represents the mean number of items recalled from other sets in Experiment 2, in all order conditions. Vertical bars represent the standard errors.

In conclusion, the results of Experiment 1 were closely replicated in Experiment 2 in the comparable conditions (cf. Table 2 and figure 4A in the main text), ruling out an alternative explanation based on the number of interfering events between the presentation and test of items in the critical set.

# **Additional Material for Experiment 3**

Experiment 3 also tested an alternative interpretation of Experiment 1 results: that the effect of the number of sets could be confounded with temporal grouping due to the presence of longer pauses between the presentation of each set in that experiment. Therefore, we excluded the longer intervals between sets in Experiment 3, with all other parameters kept identical to Experiment 1. Figure S3 represents the sequence of events in a trial of Experiment 3, using condition [33] as an example.

## **Figure S3**

## *Example of a Trial in Condition [33] in Experiment 3*

**
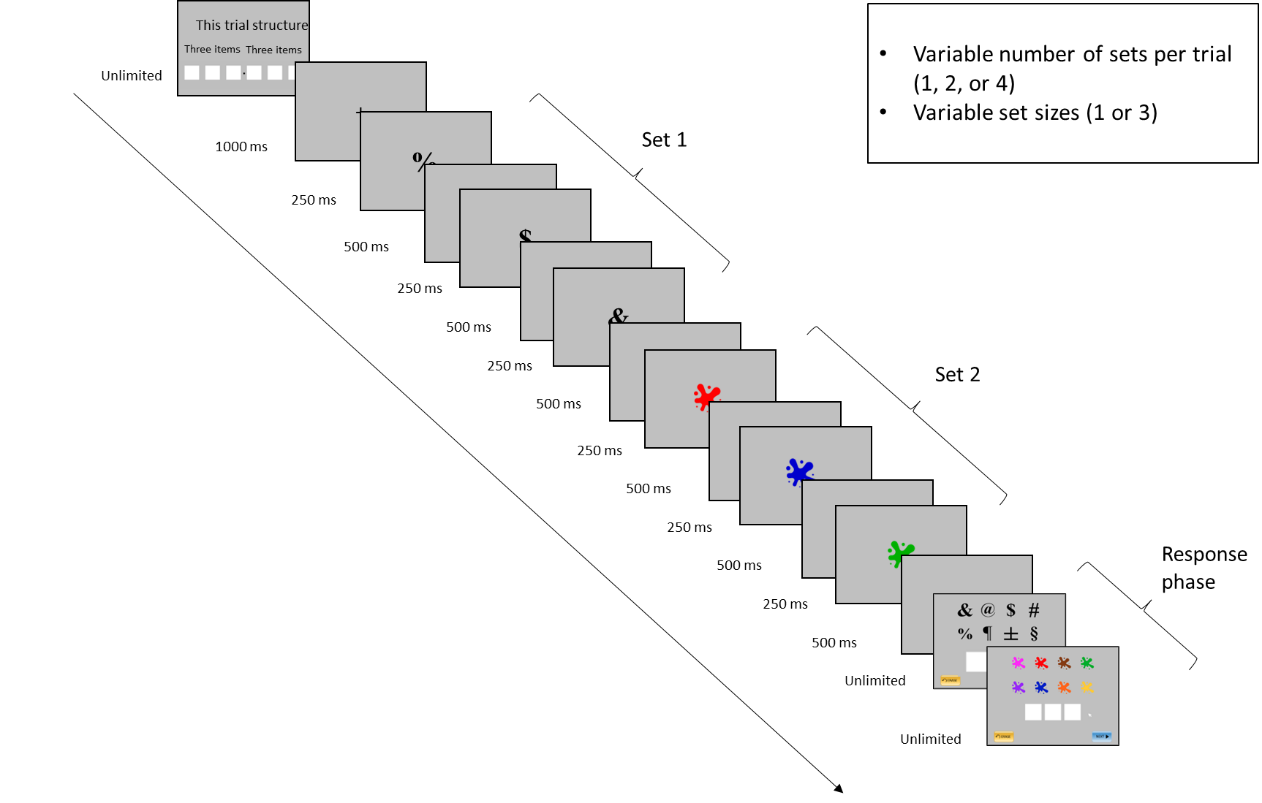
**

*Note.* The exemplified trial is condition [33], with a set of three special characters and a set of three colors. The only difference from Experiment 1 design is that the sets were presented without a longer interval between them. The figure is not to scale.

Table S2 contains the descriptive statistics of the number of items recalled from Set 1, other sets, and across sets of Experiment 3, in each experimental condition.

## **Table S2**

## *Mean Number of Items Recalled from the Critical Set A and Other Sets, per Condition of Experiment 3*

| Condition | Set 1 | Other sets | Across sets |
| --- | --- | --- | --- |
| [3] | 2.65 (0.06)  [2.53-2.78] | - | 2.65 (0.06)  [2.53-2.78] |
| [31] | 2.39 (0.8)  [2.23-2.54] | 0.76(0.03)  [0.70-0.81] | 3.14 (0.10)  [2.94-3.34] |
| [33] | 2.05 (0.08)  [1.90-2.20] | 1.42 (0.10)  [1.22-1.62] | 3.47 (0.16)  [3.14-3.79] |
| [33 same] | 1.94 (0.08)  [1.78-2.10] | 1.05 (0.09)  [0.87-1.24] | 2.00 (0.16)  [2.67-3.32] |
| [3111] | 2.04 (0.08)  [1.86-2.19] | 1.60 (0.10)  [1.40-1.79] | 3.62 (0.17)  [3.28-3.95] |
| [3333] | 1.32 (0.08)  [1.15-1.49] | 2.08 (0.20)  [1.69-2.47] | 3.40 (0.25)  [2.85-3.84] |

*Note.* Values between parentheses represent the standard errors and values between brackets represent the 95% credible intervals.

Figure S4 compares the results obtained in experiments 1 and 3, for both Set 1 (Panel A) and other sets (Panel B). For Set 1, the absence of an effect of the experiment is evident to the eye, with the same overall pattern in both experiments (Panel A). The only difference between the two experiments is the equality between conditions [33] and [3111] in Experiment 3 – evidence against an effect of the number of sets upon recall of Set 1. Recall of other sets (Panel B) was better in Experiment 1, especially in condition [3333], causing an interaction between experiment and condition.

## **Figure S4**

## *Mean Number of Items Recalled from Set 1 and Other Sets in Experiments 1 and 3*


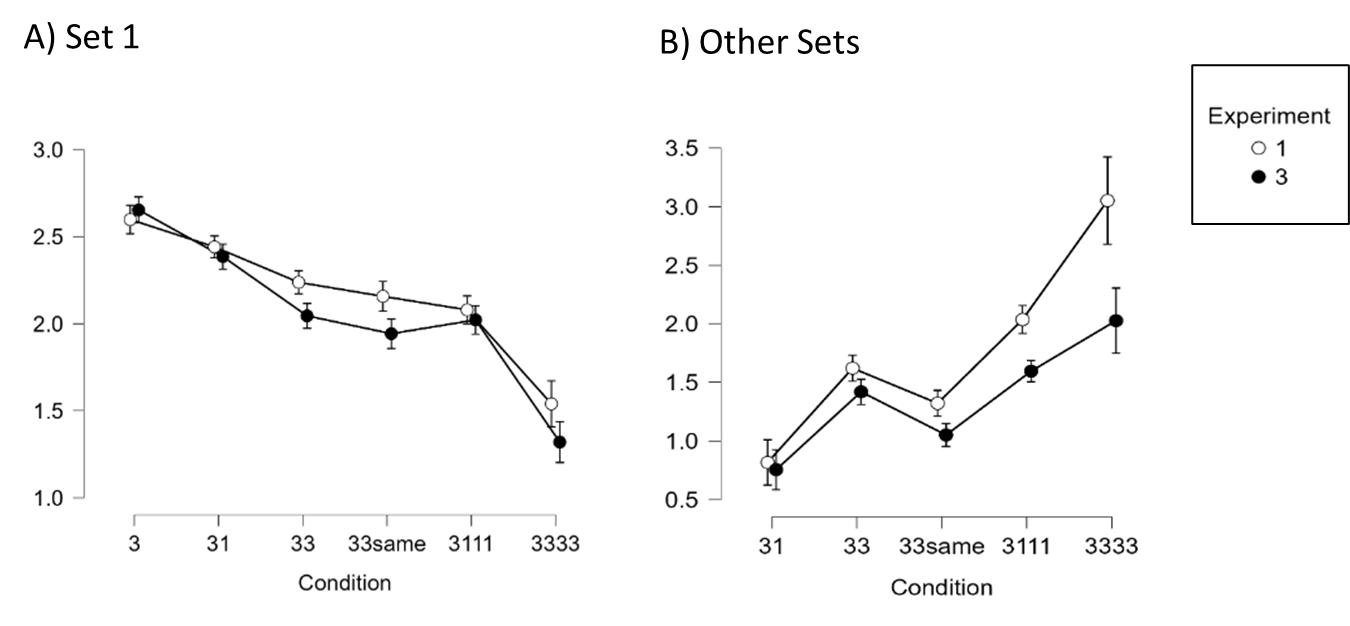


*Note.* Panel (A) shows the mean number of items recalled from Set 1. The only paired comparison between conditions that reliably differed between experiments was between [33] and [3111], according to which the advantage in [33] was abolished in Experiment 3. Panel (B) shows the mean number of items recalled from other sets. The result of all paired comparisons between conditions were replicated between experiments but performance was reliability higher in Experiment 1 (with pauses). The interaction between condition and experiment is seen in even higher recall in condition [333] in Experiment 1

# **Additional Material for Experiment 4**

Here we tested whether presenting items of the same set in adjacent serial positions was fundamental for the effect of the number of sets observed in Experiment 1. We did so by either presenting the sets in a grouped structure, with all items within a set in adjacent serial positions, or in a scrambled structure, with items within a set in non-adjacent serial positions. We chose seven variations of order in scrambled conditions. Table S3 describes and exemplifies the possible order of presentation of items in each experimental condition of Experiment 4, and Figure S5 shows an example of trial in scrambled conditions.

## **Figure S5**

## *Example of Trial in a Scrambled Condition of Experiment 4, Using Condition [33] as an Example*


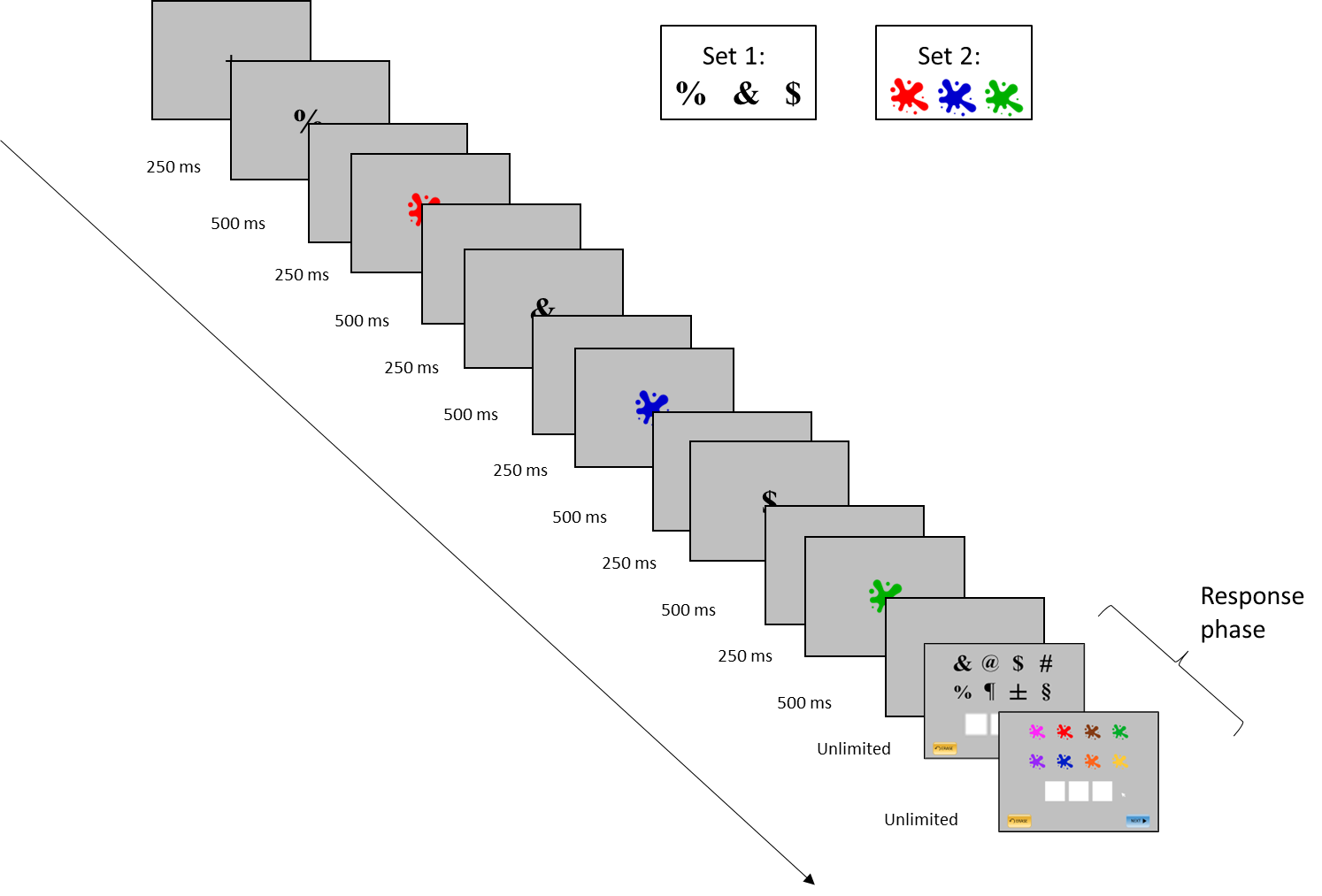


*Note.* The figure is not to scale. There was no pre-trial warning about the trial structure in Experiment 4.

## **Table S3**

## *Description of Conditions in Experiment 4.*

| Condition | |  |
| --- | --- | --- |
| Sets | Organization | Trial types |
| [3111] | Grouped | A_1_A_2_A_3_BCD |
| [3111] | Scrambled | A_1_A_2_BA_3_CD  A_1_A_2_BCA_3_D  A_1_BA_2_A_3_CD  A_1_BA_2_CDA_3_  A_1_BCA_2_A_3_D  A_1_BCA_2_DA_3_  A_1_BA_2_CA_3_D |
| [33] | Grouped | A_1_A_2_A_3_B_1_B_2_B_3_ |
| [33] | Scrambled | A_1_A_2_B_1_A_3_B_2_B_3_  A_1_A_2_B_1_B_2_A_3_B_3_  A_1_B_1_A_2_A_3_B_2_B_3_  A_1_B_1_A_2_B_2_B_3_A_3_  A_1_B_1_B_2_A_2_A_3_B_3_  A_1_B_2_B_3_A_2_B_3_A_3_  A_1_B_1_A_2_B_2_A_3_B_3_ |
| [33 same] | Grouped | A_1_A_2_A_3_A_4_A_5_A_6_ |

*Note.* Items in Set 1 are represented by the letter “A”, in Set 2 by the letter “B”, in Set 3 by the letter “C” and in Set 4 by the letter “D”. The subscripted numbers indicate that each item is unique in its feature type a given trial. The column “Trial Type” contains a description of the order of presentation of items in each condition.

Table S4 contains the descriptive statistics of the number of items recalled from Set 1, the other sets, and across sets, in each condition of Experiment 4.

## **Table S4**

## *Mean Number of Items Recalled from Set 1, Other Sets, and Across Sets, per Condition of Experiment 4*

| Condition | Set 1 | Other sets | Across sets |
| --- | --- | --- | --- |
| [3111]*_Grouped_* | 1.77 (0.10)  [1.36-1.75] | 1.20 (0.10)  [1.00-1.40] | 2.97 (0.19)  [2.27-2.94] |
| [3111]*_Scrambled_* | 1.56 (0.10)  [1.36-1.75] | 1.05 (0.08)  [0.88-1.20] | 2.60 (0.19)  [2.27-2.94] |
| [33]*_Grouped_* | 1.79 (0.11)  [1.57-2.00] | 1.08 (0.08)  [0.90-1.26] | 2.87 (0.19)  [2.50-3.24] |
| [33]*_Scrambled_* | 1.62 (0.09)  [1.44-1.80] | 0.98 (0.8)  [0.83-1.14] | 2.61 (0.16)  [2.29-2.93] |
| [33 same] | 1.70 (0.11)  [1.48-1.92] | 0.92 (0.08)  [0.76-1.09] | 2.63 (0.18)  [2.26-2.99] |

*Note.* Values between parentheses represent the standard errors and values between brackets represent the 95% credible intervals.

# **Normal Probability Plots of the Residuals (Q-Q Plots) for Aggregated Data**

Data was aggregated per participant in order to run the Bayesian ANOVAs and we have verified that the aggregated participant averages are normally distributed. This is evidenced by the QQ-plots of the residuals of participant means, which align closely with the expected normal distribution (see below). This normality supports our judgment that the standard analyses are sufficiently apt. The plots below represent the distribution of residuals of participant means in each experiment in our study. The plots were retrieved from JASP output.

## ***Experiment 1, Set 1***

## ***Experiment 1, Other Sets***

## ***Experiment 2, Critical Set A***

## ***Experiment 3, Set 1***

## ***Experiment 3, Other Sets***

## ***Experiment 4, Set 1***

## ***Experiment 4, Other Sets***
